# A mutant of *Chlamydomonas* without LHCSR maintains high rates of photosynthesis, but has reduced cell division rates in sinusoidal light conditions

**DOI:** 10.1371/journal.pone.0179395

**Published:** 2017-06-23

**Authors:** Michael Cantrell, Graham Peers

**Affiliations:** Department of Biology, Colorado State University, Fort Collins, CO, United States of America; University of Hyderabad School of Life Sciences, INDIA

## Abstract

The LHCSR protein belongs to the light harvesting complex family of pigment-binding proteins found in oxygenic photoautotrophs. Previous studies have shown that this complex is required for the rapid induction and relaxation of excess light energy dissipation in a wide range of eukaryotic algae and moss. The ability of cells to rapidly regulate light harvesting between this dissipation state and one favoring photochemistry is believed to be important for reducing oxidative stress and maintaining high photosynthetic efficiency in a rapidly changing light environment. We found that a mutant of *Chlamydomonas reinhardtii* lacking LHCSR, *npq4lhcsr1*, displays minimal photoinhibition of photosystem II and minimal inhibition of short term oxygen evolution when grown in constant excess light compared to a wild type strain. We also investigated the impact of no LHCSR during growth in a sinusoidal light regime, which mimics daily changes in photosynthetically active radiation. The absence of LHCSR correlated with a slight reduction in the quantum efficiency of photosystem II and a stimulation of the maximal rates of photosynthesis compared to wild type. However, there was no reduction in carbon accumulation during the day. Another novel finding was that *npq4lhcsr1* cultures underwent fewer divisions at night, reducing the overall growth rate compared to the wild type. Our results show that the rapid regulation of light harvesting mediated by LHCSR is required for high growth rates, but it is not required for efficient carbon accumulation during the day in a sinusoidal light environment. This finding has direct implications for engineering strategies directed at increasing photosynthetic productivity in mass cultures.

## Introduction

The natural aquatic light environment fluctuates across space and time. Light intensity follows a sinusoidal oscillation across the day with superimposed rapid fluctuations due to changes in cloud cover, wave focusing, turbidity and vertical mixing[1, 2]. These short term changes can cause light absorption to exceed the capacity of utilization leading to the generation of reactive oxygen species (ROS) such as singlet oxygen (^1^O_2_), hydrogen peroxide (H_2_O_2_), superoxide anions (O_2_^-^) and hydroxyl radicals (OH^-^). These ROS then damage surrounding proteins, pigments and lipids − impairing photosynthetic function and growth [3, 4]. Algae, plants and cyanobacteria dynamically regulate a process termed non-photochemical quenching of light energy (NPQ) to avoid excess damage while maintaining efficient photosynthesis in lower light fluxes. This balance between energy dissipation and light harvesting capacity allows photosynthetic organisms to maintain optimal fitness in diverse environmental niches.

NPQ is comprised of four components that contribute to light energy dissipation as heat [5–8]. The components are physiologically distinguished based on their time of induction and relaxation. The slowest component is photoinhibition (qI). qI occurs when the rate of damage to the PSII D1 protein exceeds the rate of its repair and is caused by absorption of light energy in excess of its utilization and NPQ capacity [9–11]. Faster forms of NPQ limit the amount of qI that occurs by protecting PSII reaction centers from over excitation. Zeaxanthin-dependent quenching (qZ) occurs on the time scale of tens of minutes and involves the pH dependent accumulation of zeaxanthin in the thylakoid membranes. Zeaxanthin is then hypothesized to enhance the probability of quenching chlorophyll excited states as heat in PSII minor antenna [12–14]. State transitions (qT) occur on the time scale of minutes and involves phosphorylation of PSII associated light harvesting complexes (LHCII) [15, 16]. Phosphorylated LHCII can then either aggregate and enter a quenched state or migrate and associate with PSI reaction centers enhancing PSI functional antenna size [17, 18]. The fastest component of NPQ is energy-dependent quenching (qE). qE is induced by luminal acidification and can be rapidly induced and relaxed in seconds to minutes [19, 20]. Each component of NPQ is thought to regulate photosynthetic function in response to different forms and lengths of environmental stress.

qE is the major component of NPQ involved in responding to dynamic increases in light intensity [21–23]. *Chlamydomonas* has been instrumental for the characterization of qE in algae. In *Chlamydomonas* three stress related light harvesting complexes (LHCSR1, 3.1 and 3.2) are required for induction of qE [21]. LHCSR mediated qE requires luminal acidification [24] and the xanthophylls zeaxanthin and lutein [25]. *Chlamydomonas* has an atypical violaxanthin de-poxidase which catalyzes the conversion of violaxanthin to zeaxanthin through the xanthophyll cycle [26]. LHCSR, unlike its functional analog in plants − PSBS, can bind chlorophylls and has limited quenching activity *in vitro* that is enhanced upon protonation in excess light[27, 28].

LHCSRs accumulate in response to excess light [21, 29] and the deprivation of CO_2_ [30, 31], sulphur [32], and iron [33]. Characterization of the qE-deficient *npq4* (Lacks LHCSR3.1 and LHCSR3.2) mutant in *Chlamydomonas* has demonstrated the importance of qE in limiting photoinhibition, ROS generation and cell death during a shift from low light to high light [21–23], but little is known about the impact that complete loss of qE has on cell growth and photophysiology under excess light conditions or in natural day/night cycles.

Here we report on our observations of the complete LHCSR knockout, *npq4lhcsr1* [34, 35]. We sought to investigate the impact of no LHCSR, and hence no qE, on photophysiology and cell growth in constant excess light and in a sinusoidal light regime that mimics natural changes in light, specifically as photosynthetically active radiation. We observed the expected reductions in photosynthetic capacity and cell physiology associated with growth in constant excess light for the mutant vs. wild type. Surprisingly, *npq4lhcsr1* did not display significant photoinhibition of photosynthesis in conditions that mimic natural light conditions and reduced daily growth rates were due to reduced cell divisions at night. We discuss the implications of these results for strategies to increase biofuel yields in industrial cultures of algae.

## Materials and methods

### Strains

The *Chlamydomonas reinhardtii* 4A+ strain (137c genetic background) was used as wild type strain. The *npq4lhcsr1* mutant was made in the 4A+ genetic background and does not produce any detectable LHCSR protein [35]. Both strains were obtained from Krishna Niyogi, University of California, Berkeley.

### Growth rates

Exponential growth rates were determined from tracking cell density for 2–3 days. Growth rates, μ, were calculated by linear regression using the exponential growth equation $\mu =\frac{\ln\left(F\right)-ln(I)}{\Delta t}$ where *F* is the final cell concentration, *I* the initial cell concentration, μ is the specific growth rate (d^-1^) and *t* time in days [36]. Cell densities were determined using the Accuri C6 flow cytometer (BD). Samples were passed through a 30 μm pre-filter (MACS Miltenyi, 130-041-407) prior to each run to remove debris. Each count was performed for 60 seconds using a flow rate of 35 μl min^-1^ and a core size of 14 μm. *Chlamydomonas* cells were identified as chlorophyll autofluorescence particles via excitation and emission at 640 and 675 ± 25 nm, respectively.

### Culture conditions

All experiments were performed on axenic, photoautotrophically grown cultures using TP media at 25°C [37]. Each experiment started with an inoculation of between 10,000–50,000 cells ml^-1^ from a stock culture acclimated for at least 10 generations in their respective light environment. Cells for the continuous light experiments were grown in 250 ml glass Erlenmeyer flask containing 100 mL of media and shaken at 150 RPM. Continuous light cultures were grown under 50, 400 or 860 μmol photons m^-2^ s^-1^ of Photosynthetically Active Radiation (PAR light supplied by cool white fluorescent bulbs (Phillips, F17T8/TL841). Light fluxes were measured using a 2pi, LICOR LI-250A light sensor.

**Fig 1:**
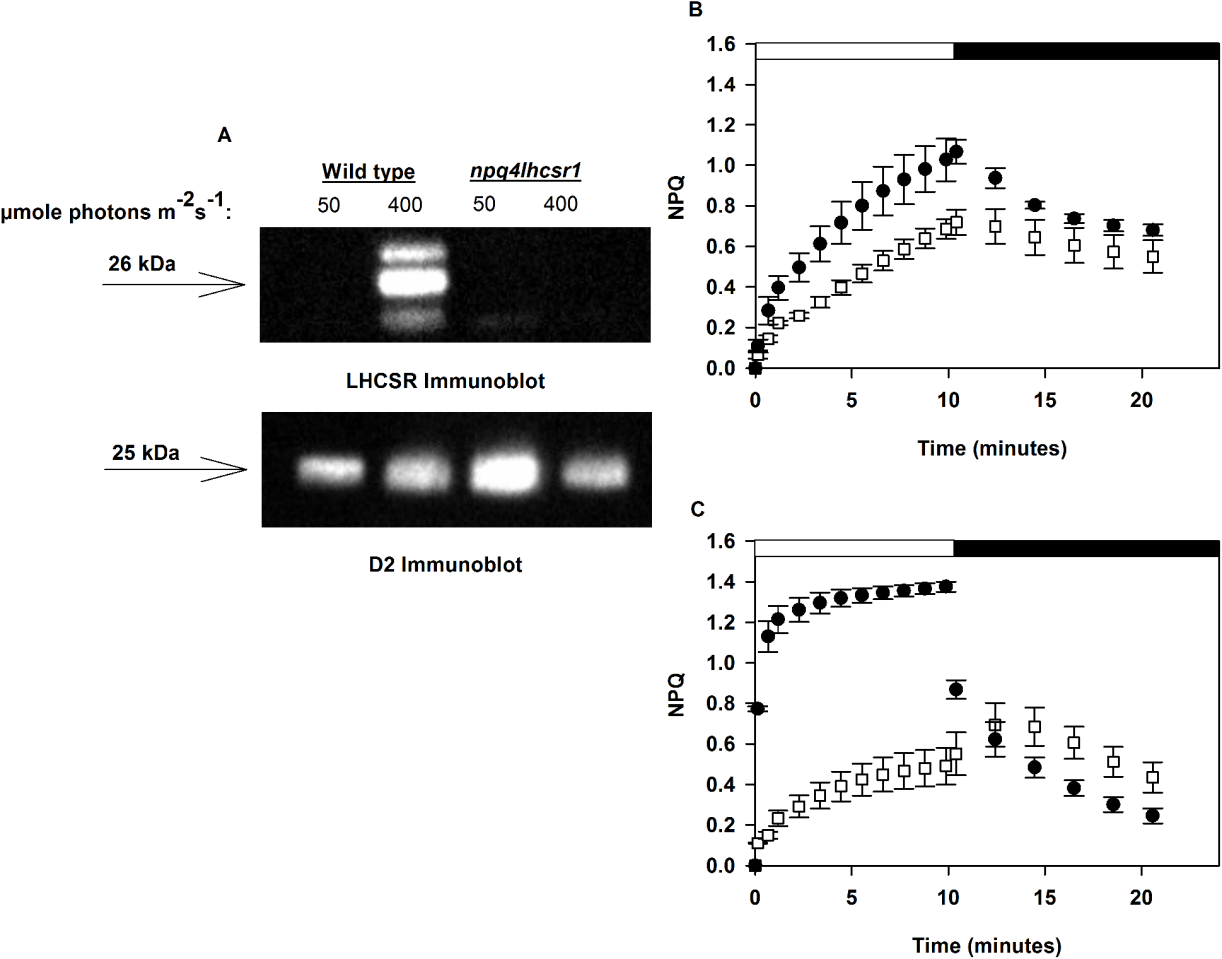
*npq4lhcsr1* lacks detectable LHCSR and rapidly reversible NPQ. (A) Immunoblot analysis of LHCSR and D2 protein of the photosystem II reaction center from cultures acclimated to a continuous irradiance of 50 μmol photons m^-2^ s^-1^ and 400 μmol photons m^-2^ s^-1^. 0.77 nmol of chlorophyll was loaded in each lane. (B-C) Wild type (black circles) and the *npq4lhcsr1* (white squares) mutant cultures acclimated to 50 μmol photons m^-2^ s^-1^ (B) and 400 μmol photons m^-2^ s^-1^ (C) when exposed to a red actinic light level of 2005 μmol photons m^-2^ s^-1^ (white bar) followed by 10 minutes darkness (black bar) and far red light illumination to re-associate LHCII with PSII (state 1 transition) by preferentially driving PSI charge separation. Data represents means ± s.d. (n = 3).

Growth and physiology in sinusoidal light conditions were characterized using a Phenometrics ePBR with a custom, autoclavable glass culture vessel [38, 39]. Cells were grown in 500 mL of TP mixed by a stir bar running at 500 RPM and sparged with 0.45 μm filtered air supplied at 1 L min^-1^. Cultures were illuminated with a white LED light programmed to follow a 12:12 light:dark cycle. Light intensity was programmed to either follow a square wave function with light intensity equaling 2134 μmol photons m^-2^ s^-1^ of photosynthetically active radiation (PAR) or to follow a sinusoidal light pattern peaking at 2134 μmol photons m^-2^ s^-1^ of PAR at the top of a 24 cm water column and 370 μmol photons m^-2^ s^-1^ at the bottom of the culture 6 hours after dawn (4pi Walz ULM-500 light meter, Fig 1B). The light intensity for either position can be approximated for *t* hours after dawn as follows:

$$Lightflux\;(t)=A_{max}\cdot \sin(\pi ∙t\cdot \frac{1}{12})$$

*where A*_*max*_ = 2134 *or* 370 μmol photons m^−2^ s^−1^, and t = hours after dawn.

See Jallet et al. (2016) for a full description and diagram of the ePBR culturing set up and Lucker et al. (2014) for the light spectra associated with these LEDs vs cool white fluorescent bulbs [38, 39].

### Analyses of chlorophyll fluorescence parameters in steady state

Chlorophyll fluorescence measurements of *Chlamydomonas* were performed using a Dual-PAM (Walz). All measurements were made using a blue measuring light (460 nm, intensity setting 4) and red actinic light (620 nm). 5 ml of culture in exponential growth phase (ranging from 450,000 cells ml^-1^ to 750,000 cells ml^-1^) was agitated in a 50 mL beaker for 20 minutes prior to each measurement. Cells were then collected by gently filtering onto a 13 mm diameter glass fiber filter (Millipore, AP2501300) that was then placed in the Dual-PAM’s leaf clip [21]. PSII quantum efficiency was measured as $\frac{F_{m}-F_{o}}{F_{m}}$, where *F*_*m*_ is the maximum PSII fluorescence obtained with a red saturating pulse (620 nm, 600 ms duration, 10,000 μmol photons m^-2^ s^-1^) and *F*_*o*_, the minimum fluorescence obtained after 10 minutes of far red light (intensity setting 10) to ensure a state 1 transition [21]. Cells were then illuminated with either 600 μmol photons m^-2^ s^-1^ or 2005 μmol photons m^-2^ s^-1^ for 10 minutes. Saturating pulses were applied every minute during illumination. The actinic light was turned off, and the cells were re-illuminated with far red light and saturating pulses were applied every 2 minutes for 10 minutes. NPQ was calculated as $NPQ=\frac{F_{m}^{\prime }-F_{m}}{F_{m}^{\prime }}$ where *F*_*m*_*’* is the maximum fluorescence measured in the light-adapted state (during or after actinic light illumination). Closed PSII reaction centers were calculated as 1-qL, assuming the lake model [40, 41]. $qL=\frac{(F_{m}^{\prime }/F)}{\left(F_{m}-F_{o}\right)}∙\frac{F_{o}^{\prime }}{F}$ with *F* representing fluorescence in the illuminated state and *Fo’*, representing an approximation of the minimum fluorescence obtainable in an illuminated state, is calculated as *Fo’* = $\frac{F_{o}}{((F_{m}^{\prime }-F_{m}/F_{m})+F_{o}/F_{m})}$ [40, 41].

### Simultaneous measurements of chlorophyll fluorescence and oxygen evolution

Cultures in late exponential (450,000 cells ml^-1^ to 750,000 cells ml^-1^) were collected by centrifugation at 1500 x g for 5 minutes at 25°C. The supernatant was discarded. The pellet was then re-suspended in TP media supplemented with freshly prepared NaHCO_3_ to final concentration of 5 mM. Cell density was adjusted to 4 ± 1 μM chlorophyll. One ml of these cells was transferred into a homemade 1 cm diameter cylindrical quartz cuvette with a magnetic stir bar. Cells were dark acclimated for 15 minutes then sealed in the cuvette using a custom 3D-printed plastic stopper fitted with a FireSting dark-coated robust probe (OXROB10) [39]. Dark respiration rates were recorded for 5 minutes before turning on the Dual-PAM far red light (intensity setting 20) for 10 minutes to induce a state 1 transition [21]. An initial red saturating pulse (620 nm, 600 ms, 10,000 μmol photons m^-2^ s^-1^) was used to measure maximum PSII quantum efficiency (as described above) followed by a rapid light curve (RLC) of increasing 60-second actinic red light steps punctuated by saturating pulses. The assayed actinic light steps included 8, 19, 33, 56, 80, 106, 143, 191, 250, 323, 407, 505, 635, 783, 1203, 1850 μmol photons m^-2^ s^-1^ (measured with a 4pi Walz ULM-500 light meter). Gross oxygen evolution rates were determined based on the sum of the net oxygen evolution rates over the last 45 seconds of each light step and the absolute value of the dark respiration rates. The maximum oxygen evolution rate (P_max_), the light limited slope (α) and the irradiance at saturation (I_K_) was determined by fitting oxygen evolution rates (nmol O_2_ cell^-1^ min^-1^) vs irradiance to the exponential difference equation described by Platt et al. (1980) using the curve fitting tool described in Ritchie (2008) [42, 43].

### Dark respiration rates at night in sinusoidal light cultures

Dark respiration rates we measured ex-situ, using the apparatus described above, on late exponential cultures (ranging from 450,000 cells ml^-1^ to 1,500,000 cells ml^-1^). Respiration rates were measured immediately after sampling at 20 minutes before and after the dark period and 0.5, 1.5, 3, 6, 9, 10.5 and 11.5 hour after dusk.

### Chlorophyll fluorescence parameters during growth in sinusoidal conditions

PSII fluorescence induction profiles across the sinusoidal light period were measured using a Fluorescence Induction and Relaxation Fluorometer (FIRe, Satlantic) using a blue (450 nm with a 30 nm band-width) LED to excite chlorophyll *a*. A saturating (5 x 10^4^ μmol photons m^-2^ s^-1^) single turnover flash (80 μs) and multiple turnover flash (20 ms) were used to calculate the effective quantum yield of PSII (*F*_*v*_/*F*_*m*_), the relative functional antenna size of PSII (sigma_PSII_) and the re-oxidation kinetics of the quinone Q_B_ (τ) as described in Kolber et al. 1998 [44]. *Chlamydomonas* cells (600,000 cells ml^-1^ to 1,000,000 cells ml^-1^) were collected from the ePBR photobioreactors and measured within 1 minute using a FIRe fluorometer (Satlantic). We used the MATLAB software and the FIReWORX script (Audrey Barnett, http://sourceforge.net/project/fireworx/) with instrument specific light calibrations factors to extract fluorescence parameters, *F*_*o*_ and *F*_*m*,_ and fit the fluorescence induction and relaxation curves generated.

### Lipid peroxides

Lipid peroxidation was estimated by measuring the formation of thiobarbituric acid-reactive substances (TBARS) [45, 46]. 1 x 10^7^ cells were harvested during exponential growth phase (600,000 cells ml^-1^ to 1,000,000 cells ml^-1^). We added 0.01% (w/v) butylated hydroxytoluene (BHT, Sigma-Aldrich) to each sample prior to freezing at -80°C to arrest lipid peroxidation. Thawed cell samples were pelleted at 3220 x g for 5 minutes at 4°C after the addition of 0.01%, final concentration, TWEEN 20 (Sigma-Aldrich). Cell pellets were re-suspended in 1 ml ice-cold 80:20 (v/v) ethanol:water and lysed using sonication for 10 seconds (Q55 QSonica probe sonicator, 50% duty cycle). After sonication, 750 μL of homogenate was added to a 9 ml glass screw top test tube with 750 μL of either a +TBA solution (20.0% trichloracetic acid, 0.01% butylated hydroxytoluene, and 0.65% 2-thiobarbituric acid, all w/v, Sigma-Aldrich) or a -TBA solution. Samples were then heated to 95°C in a water bath for 25 minutes and then cooled to room temperature. One mL of this solution was transferred to a centrifuge tube and clarified by centrifugation at 10,000 x g for 5 minutes at room temperature. TBARS in the supernatant was determined by absorbance at 532 nm with a correction for nonspecific absorbance at 440 nm and 600 nm and normalized per 1 x 10^7^ cells [46].

### Pigment concentration

Cells were initially harvested as described in the simultaneous measurements of chlorophyll fluorescence and oxygen evolution. TWEEN 20 (0.01% final concentration, Sigma-Aldrich, p7949) was added to 1 mL of culture and the cells were centrifuged at 10,000 x g for 10 minutes at 4°C. The supernatant was removed. Chlorophylls were then extracted for 10 minutes in 80% buffered acetone with 50 mM HEPES, pH 7.5 and then cell debris was removed by centrifugation at 10,000 x g for 5 minutes [47]. Chlorophyll *a* and *b* concentrations were determined by spectrophotometry using established extinction coefficients [47].

### Cell volumes

*Chlamydomonas* cell volumes were determined assuming a prolate spheroid shape [48]. Cell dimensions were determined from at least 100 size-calibrated cell images from a Leica DM5500 B microscope using imageJ [49].

### Total organic carbon

Total organic carbon (TOC) per cell were determined using a Shimadzu TOC-L Laboratory Total Organic Carbon analyzer. 9.5 x 10^6^ to 15 x 10^6^ cells were harvested from low density cultures (ranging from 450,000 cells ml^-1^ to 1,600,000 cells ml^-1^) by centrifugation (at 3,220 x g for 5 minutes at 25°C) in acid washed (10% HCl) 50 ml conical centrifuge tubes. Cell pellets were rinsed twice with an isotonic (0.523 mM NaCl) solution, re-suspended in 20 ml of 0.523 mM NaCl and then stored at -80°C. Samples were thawed on the day of analysis and 5 x 10^6^ cells were diluted with water (18.2 MΩ) to a final volume of 40 ml in TOC glass vials (Sigma-Aldrich). Sample blanks were prepared using an equal volume of isotonic salt solution and water. Measurements and a calibration curves were made for Total Carbon (TC), and Inorganic Carbon (IC). Blank and internal controls with known TC or IC were tested throughout an individual set as a control. Each sample was measured sequentially in technical duplicate using a 100 μl injection volume for TC and IC measurements. TOC per cell were calculated based on the manufacturer’s instructions (Shimadzu Application News no. 049) as follows:

$${TC}_{cells}=\frac{{TC}_{sample}–{TC}_{blank}}{Cells\;in\;diluted\;sample}$$

$${IC}_{cells}=\frac{{IC}_{sample}–{IC}_{blank}}{Cells\;in\;diluted\;sample}$$

$${TOC}_{cells}={IC}_{sample}–{IC}_{blank}$$

Carbon accumulation across the day was calculated by subtracting TOC per cell at dawn from TOC per cell at dusk within each biological replicate.

### Cell division frequency measurements

Division frequency and average division number under sinusoidal conditions was determined as previously described [50], but with some modifications. 300 μl of each culture were harvested at the start of the dark period and plated on TP agar plates (1.5%). Plates were incubated for 24–30 hours in the dark and scored by counting 300–400 cell clusters with each cluster representing the progeny from one mother cell after dark incubation. The number of cells per cluster was used to determine the division number, n, for the multiple-fission events occuring during the night [51].

### Immunoblot analysis

Cells were harvested in exponential growth phase (ranging from 450,000 cells ml^-1^ to 750,000 cells ml^-1^) by centrifugation (1,800 x g for 4 minutes at 25°C). Samples were then prepared and ran on pre-cast Novex 10–20% tris-glycine gels (EC6135BOX) as described in Peers et al. 2009 with the following modifications [21]. Membranes were blocked overnight with 3% milk in TBS with 1% TWEEN 20 (Sigma-Aldrich, P7949) and then incubated with anti-LHCSR polyclonal antibody [52], anti-D2 antibody (PsbD, Agrisera #AS06146), anti-PsaC antibody (Agrisera #AS10939), anti-Lhcb2 (Agrisera #AS01003) diluted 1:2,000, or anti-ATP-B (Agrisera #AS05085) diluted 1:5,000, in a 0.5% milk TBS solution. Membranes were incubated with each respective antibody for one hour and then rinsed four times for 5 minutes. Band detection was performed using SuperSignal West Femto Maximum Sensitivity Substrate (Thermo Scientific, #34095) according to the manufacturer’s protocol. 0.77 nmol total chlorophyll was loaded for immunoblots of LHCSR. 0.075 nmol total chlorophyll was loaded for immunoblots of D2 and PsaC. 0.2 nmol total chlorophyll was loaded for Lhcb2 immunoblots. Protein abundance was determined by the band intensity using the imageJ software, version 1.48 (http://imagej.nih.gov/ij/). Relative abundances for D2, PsaC and Lhcb2 are based on an in gel dilution series (25, 50, 100, 150% of WT). Relative abundance was normalized within samples to ATP-B.

### Statististics

Measurements were made on at least three independent cultures except for cell division frequency measurements. An un-paired Student’s t-test was performed to compare *npq4lhcsr1* to wild type (SigmaStat).

## Results

### Growth rates of wild type and the *npq4lhcsr1* mutant under continuous and sinusoidal light regimes

*Chlamydomonas* cultures were acclimated for at least 10 generations to constant light conditions before physiological characterization. The *npq4lhcsr1* mutant obtained did not have any detectable LHCSR protein expression (Fig 1A) and no rapidly inducible NPQ when acclimated to 400 μmol photons m^-2^ s^-1^ (Fig 1C). There was also no rapidly inducible NPQ observed in either wild type and *npq4lhcsr1* cells acclimated to 50 μmol photons m^-2^ s^-1^.

**Fig 2:**
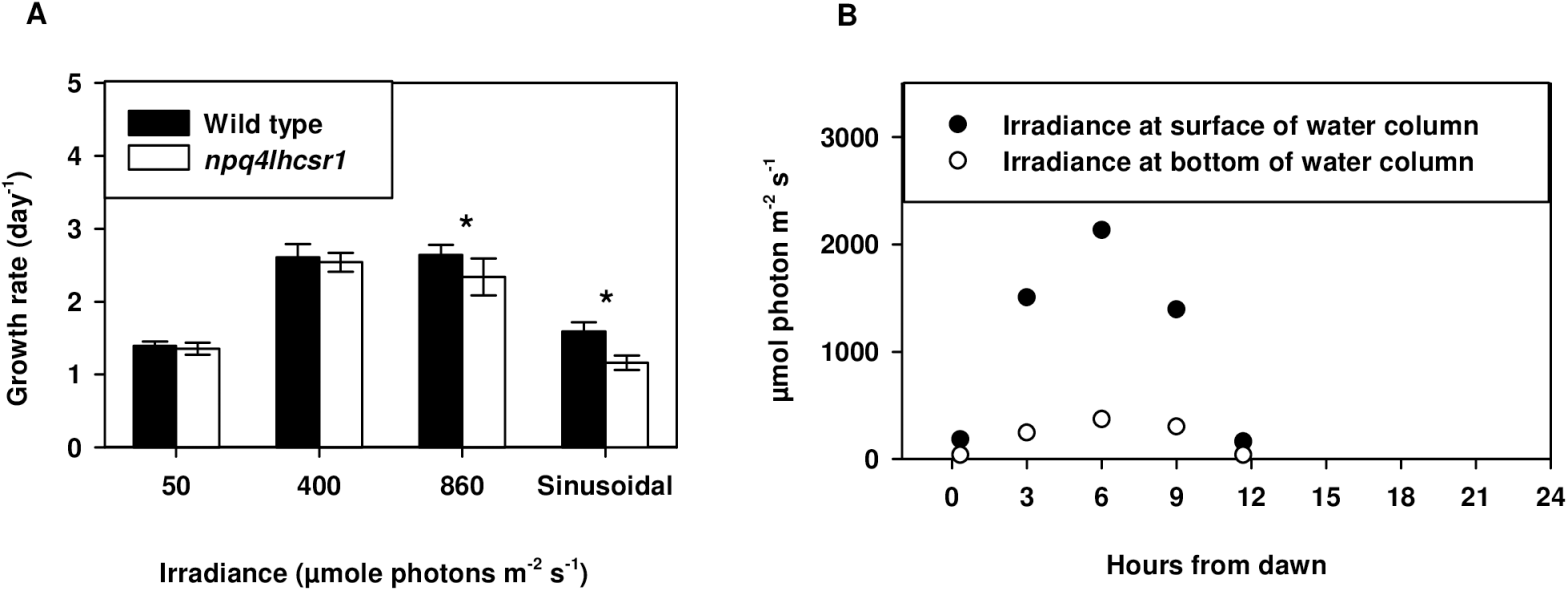
*npq4lhcsr1* growth rate under continuous and sinusoidal conditions. (A) *Chlamydomonas* exponential growth rates determined by cell counts collected over 2–3 days of growth. Symbols (*) represent significant differences from wild type within light acclimated states based on an un-paired t-test (p < 0.05). Data represents means + s.d. (n = 7–9) (B) Light levels measured across the 12:12 light dark cycle used for the sinusoidal light regime.

We tested the effects of qE loss on cell physiology using five light regimes. To test if qE is required for rapid growth in continuous excess light, cultures were grown under continuous irradiance of 50, 400 and 860 μmol photons m^-2^ s^-1^. Under continuous irradiances wild type growth rate was highest at 400 and 860 μmol photons m^-2^ s^-1^ (Fig 2A). However, the measured growth rates were reduced by 11% in *npq4lhcsr1* relative to wild type at the highest irradiance (p<0.05). To test the requirement of qE for growth in a sinusoidal light regime, we grew cells in a simulated “natural” light regime with a 12:12 light:dark cycle and a peak light intensity of 2134 μmol photons m^-2^ s^-1^ at the top of the water column (Fig 2B). We note that the light gradient found in the ePBR system resulted in light environments that rapidly changed (<10s) between the measured surface and bottom irradiances (Fig 2B). Growth in this sinusoidal light regime reduced the daily growth rate of *npq4lhcsr1* to 23% relative to wild type (Fig 1A). However, strains grown in 12 hours 2134 μmol photons m^-2^ s^-1^ light, 12 hours dark in a ePBR grew at the same rate (S4 Fig).

### Chlorophyll content of wild type and *npq4lhcsr1* under continuous and sinusoidal light regimes

Wild type cultures reduced their total chlorophyll content with increasing irradiances under continuous light acclimated conditions (Table 1). This ranged from 1.25 ± 0.09 fmol chlorophyll cell^-1^ at 50 μmol photons m^-2^ s^-1^ to 0.27 ± 0.05 fmol chlorophyll cell^-1^ under 850 μmol photons m^-2^ s^-1^ representing a 78% reduction. Wild type cells grown in the sinusoidal regime and harvested 6 hours after dawn contained 1.15 fmol chlorophyll cell^-1^.

**Table 1:** Effects of acclimation state on *npq4lhcsr1* physiology.

	Units	50 μmol photons m^-2^ s^-1^	
		wild type	*npq4lhcsr1*
Chlorophyll *a* + *b*	fmol cell^-1^	1.25 ± 0.09	1.09 ± 0.15
Chlorophyll *a*	fmol cell^-1^	0.92 ± 0.06	0.79 ± 0.10
Chlorophyll *a*:*b*	mol:mol	2.76 ± 0.04	2.66 ± 0.21
	Units	400 μmol photons m^-2^ s^-1^	
		wild type	*npq4lhcsr1*
Chlorophyll *a* + *b*	fmol cell^-1^	0.57 ± 0.03	0.64 ± 0.09
Chlorophyll *a*	fmol cell^-1^	0.43 ± 0.03	0.47 ± 0.07
Chlorophyll *a*:*b*	mol:mol	3.02 ± 0.09	2.69 ± 0.07 *
	Units	860 μmol photons m^-2^ s^-1^	
		wild type	*npq4lhcsr1*
Chlorophyll *a* + *b*	fmol cell^-1^	0.27 ± 0.05	0.31 ± 0.05
Chlorophyll *a*	fmol cell^-1^	0.20 ± 0.04	0.22 ± 0.03
Chlorophyll *a*:*b*	mol:mol	2.89 ± 0.28	2.51 ± 0.10
	Units	Sinusoidal light regime	
		wild type	*npq4lhcsr1*
Chlorophyll *a* + *b*	fmol cell^-1^	1.15 ± 0.19	1.30 ± 0.18
Chlorophyll *a*	fmol cell^-1^	0.89 ± 0.15	0.98 ± 0.13
Chlorophyll *a*:*b*	mol:mol	3.49 ± 0.08	3.07 ± 0.05*

Data represents the mean ± s.d. (n = 3).

Symbols (*) represent significant differences from wild type within light acclimated states based on an un-paired t-test (p < 0.05).

The chlorophyll *a*:*b* ratio can be used to assess *Chlamydomonas’s* photoacclimation response. Chlorophyll *b* is only found in the photosynthetic antenna proteins and thus higher ratios of chlorophyll *a*:*b* indicate a reduction in antenna size [1]. At 50 μmol photons m^-2^ s^-1^ the chlorophyll *a*:*b* ratio was statistically identical between wild type and *npq4lhcsr1* (p > 0.05). We observed some reductions in the ratio between the mutant and wild type at higher light levels, but this was only statistically significant in cultures acclimated to 400 μmol photons m^-2^ s^-1^ (Table 1). 6 hours after dawn in the sinusoidal light regime, the *npq4lhcsr1* chlorophyll *a*:*b* ratio was significantly reduced compared to wild type cells.

### Photosynthetic responses of wild type and *npq4lhcsr1*

**Fig 3:**
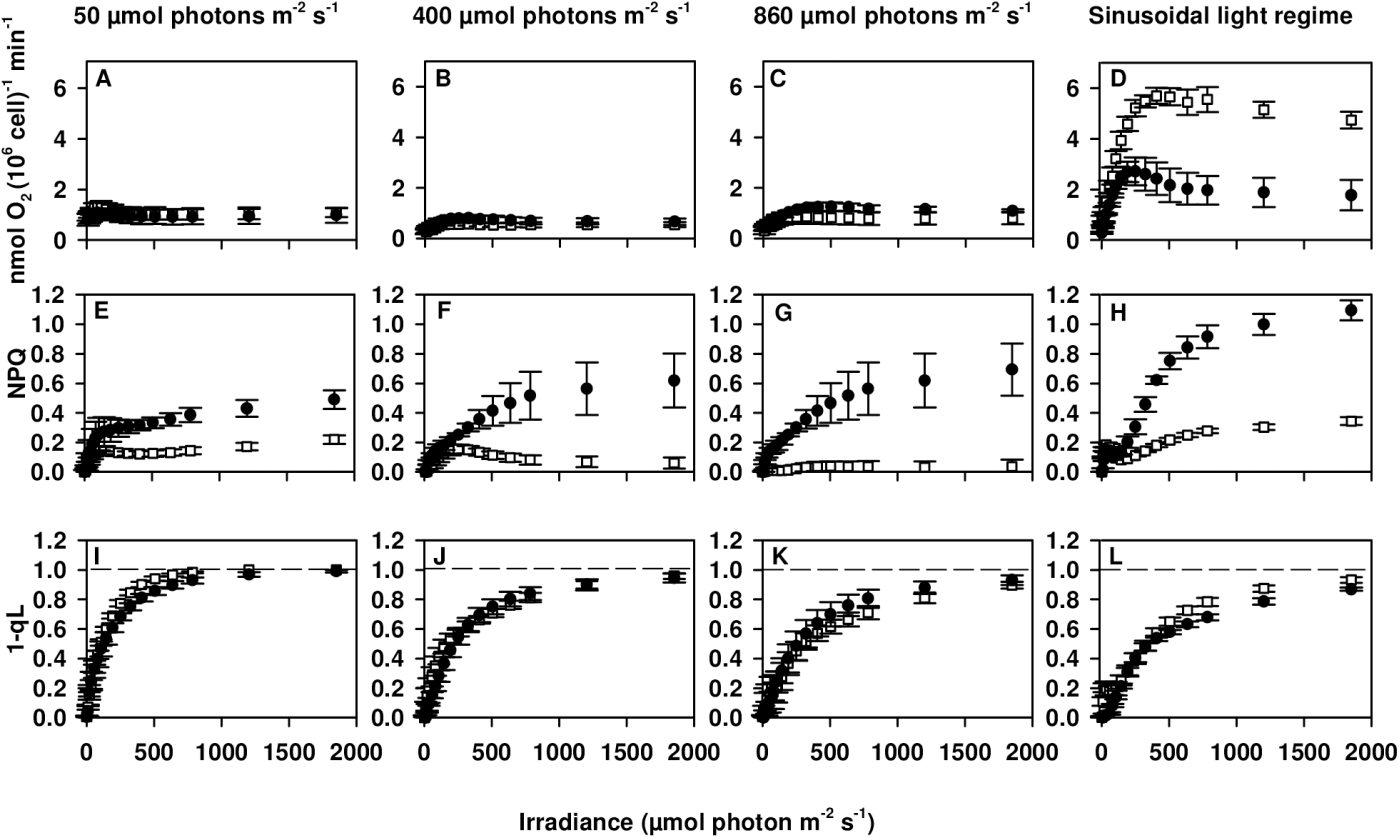
Photosynthesis vs Irradiance (P vs I) curves for wild type and *npq4lhcsr1* acclimated to different light regimes. Wild type (black circles) and the *npq4lhcsr1* mutant (white squares) acclimated to either 50, 400, 860 μmol photons m^-2-^ s^-1^ or a sinusoidal light regime (data collected 6 hours after dawn) were exposed to consecutive, increasing intensities of red light. Oxygen concentrations and PAM fluorescence were measured simultaneously. Data represent means ± s.d. (n = 3–6).

Significant reductions in maximum PSII quantum yield (*F*_*v*_*/F*_*m*_) for *npq4lhcsr1* cells grown under 400 and 860 μmol photons m^-2^ s^-1^ correlated with a significant reduction in the maximum oxygen evolution rate per cell (P_max_) only under 860 μmol photons m^-2^ s^-1^ (Table 2). I_K_ and α remained the same between each strain in our excess light conditions. NPQ capacity in wild type increased between high and low light grown cells with similar levels of maximum NPQ observed between cultures acclimated to continuous irradiances of 400 and 860 μmol photons m^-2^ s^-1^ (Fig 3E–3G). NPQ capacity was reduced in *npq4lhcsr1* compared to wild type in all conditions. NPQ capacity was found to be rapidly reversible in the dark for wild type cells, suggesting most of the NPQ was due to qE. In the *npq4lhcsr1* strain little of the observed NPQ was reversible in the dark, suggesting photoinhibition (qI). These results are shown in S1 Fig.

**Table 2:** Effects of acclimation state on *npq4lhcsr1* photophysiology.

Parameters	50 μmol photons m^-2^ s^-1^	
	wild type	*npq4lhcsr1*
Photosystem II efficiency (*F*_*v*_*/F*_*m*_)	0.61 ± 0.02	0.59 ± 0.01
Pmax^a^	ND	ND
α^b^	ND	ND
I_K_^c^	ND	ND
Dark Respiration^d^	8.4 x 10^−7^ ± 3.1 x 10^−7^ *	1.1 x 10^−6^ ± 1.3 x 10^−7^ *
Parameters	400 μmol photons m^-2^ s^-1^	
	wild type	*npq4lhcsr1*
Photosystem II efficiency (*F*_*v*_*/F*_*m*_)	0.54 ± 0.01	0.47 ± 0.03 *
Pmax^a^	7.9 x 10^−7^ ± 1.5 x 10^−7^	6.5 x 10^−7^ ± 1.5 x 10^−7^
α^b^	3.2 x 10^−2^ ± 0.3 x 10^−2^	2.2 x 10^−2^ ± 0.8 x 10^−2^
I_K_^c^	299 ±35	313 ± 84
Dark Respiration^d^	4.3 x 10^−7^ ± 1.2 x 10^−7^	4.6. x 10^−7^ ± 6.5 x 10^−8^
Parameters	860 μmol photons m^-2^ s^-1^	
	wild type	*npq4lhcsr1*
Photosystem II efficiency (*F*_*v*_*/F*_*m*_)	0.46 ± 0.03	0.31 ± 0.14 *
Pmax^a^	1.3 x 10^−6^ ± 1.3 x 10^−7^	9.0 x 10^−7^ ± 1.7 x 10^−7^ *
α^b^	3.1 x 10^−2^ ± 1.0 x 10^−2^	2.1 x 10^−2^ ± 0.5 x 10^−2^
I_K_^c^	513 ± 143	439 ± 120
Dark Respiration^d^	6.9 x 10^−7^ ± 5.1 x 10^−8^	5.1 x 10^−7^ ± 1.3 x 10^−7^
Parameters	Sinusoidal light regime	
	wild type	*npq4lhcsr1*
Photosystem II efficiency (*F*_*v*_*/F*_*m*_)	0.66 ± 0.01	0.62 ± 0.02 *
Pmax^a^	2.7 x 10^−6^ ± 3.0 x 10^−7^	5.7 x 10^−6^ ± 3.8 x 10^−7^ *
α^b^	3.4 x 10^−2^ ± 0.5 x 10^−2^	2.4 x 10^−2^ ± 0.3 x 10^−2^ *
I_K_^c^	380± 102	711 ± 60 *
Dark Respiration^d^	5.4 x 10^−7^ ± 1.2 x 10^−7^	9.16 x 10^−7^ ± 1.7 x 10^−7^ *

^a^Pmax (nmole O_2_ evolved min^-1^ cell^-1^)

^b^α ((nmole O_2_ evolved min^-1^ cell^-1^)/(μmol photons per m^2^))

^c^I_K_ (μmol photons per m^2^) and

^d^Dark respiration (nmol O_2_ consumed cell^-1^ min^-1^) were collected as described in the materials and methods. Low light acclimated samples had insufficient points for the accurate calculation of Pmax, α and I_K_ (not determined—ND). Values determined for sinusoidal light regime reflect samples measured 6 hours after dawn. Data represents the mean ± s.d. (n = 3–6).

Symbols (*) represent significant differences from wild type within light acclimated states based on an un-paired t-test (p < 0.05).

There was no statistically significant change in the 1-qL parameter between the two strains (Fig 3I–3L). 1-qL appeared to saturate at lower light intensities for cells grown at 50 vs 400 and 860 μmol photons m^-2^ s^-1^. Unfortunately, the small number of data points collected at low light fluxes did not permit the calculation of P_max_, I_k_ or α parameters for cells grown at 50 μmol photons m^-2^ s^-1^.

We characterized the photophysiology of cells grown in sinusoidal light conditions as well. Measurements taken 6 hours into the light period indicated only small reductions in the maximum PSII quantum yield (*F*_*v*_*/F*_*m*_, Table 2) in the mutant compared to the wild type. *npq4lhcsr1* displayed a significant increase in P_max_ and I_K_ and a decreased α compared to wild type (Table 2). NPQ capacity was reduced in *npq4lhcsr1* compared to wild type and there was no change in the 1-qL parameter between the two strains (Fig 3).

**Fig 4:**
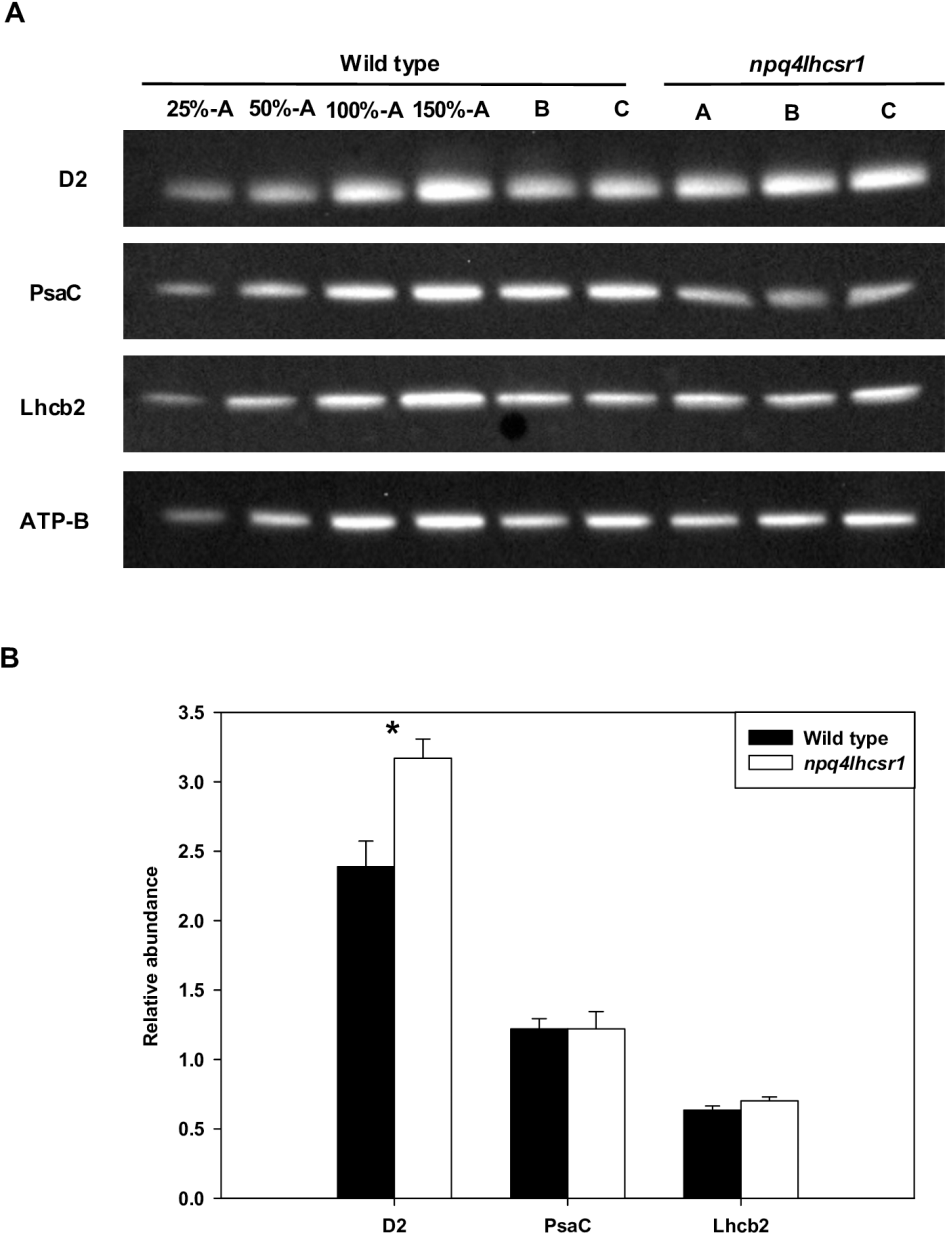
Protein abundance of D2, PsaC and Lhcb2 6 hours after dawn in sinusoidal cultures. (A) Immunoblot detection of D2, PsaC, and Lhcb2 for wild type and *npq4lhcsr1*. Lanes were loaded on an equal chlorophyll basis with biological replicates for wild type and *npq4lhcsr1* shown as A, B, and C. (B) Results from comparative densitometry of relative protein abundance normalized to ATP-B content. Data represent means ± s.d. (n = 3). Symbols (*) represent significant differences between mutant and wild type based on an un-paired t-test (p < 0.05).

Immunoblot based quantification of the PSII subunit D2, the PSI subunit PsaC and the PSII associated light harvesting complex Lhcb2 was also used to assess differences in photosynthetic architecture during growth in sinusoidal light. Relative protein content was normalized to the abundance of the beta subunit of ATP synthase (ATP-B). *npq4lhcsr1* displayed significantly more D2 subunit relative to wild type, suggesting an increase in the overall PSII:PSI ratio (Fig 4B). The relative abundance of Lhcb2 did not differ between the mutant and wild type.

**Fig 5:**
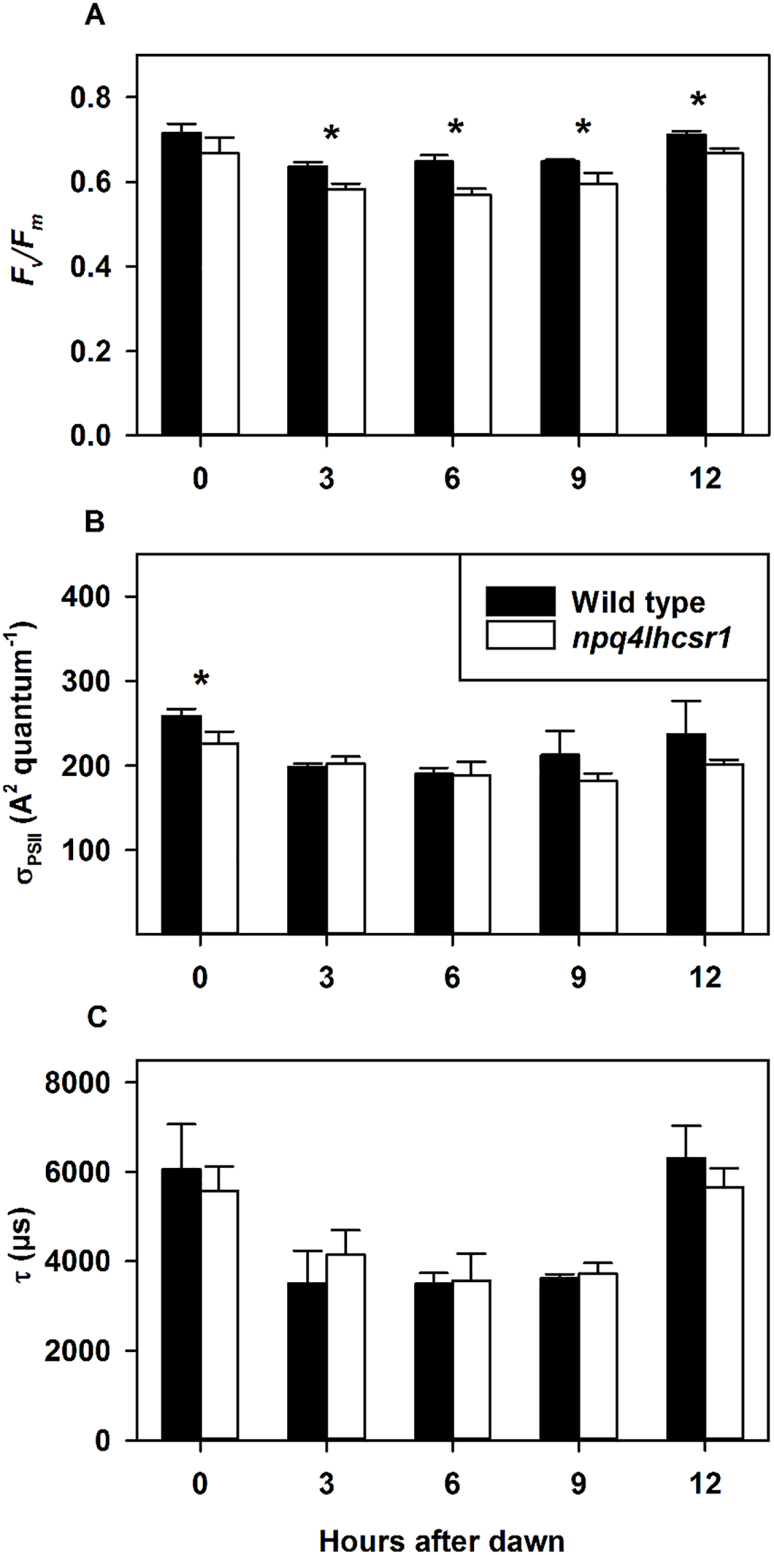
Photophysiology of photosystem II during growth in a sinusoidal light regime. (A) Effective quantum yield (*F*_*v*_*/F*_*m*_). (B) Functional antenna size (sigma_PSII_, A^2^ quantum^-1^). (C) Q_B_ re-oxidation kinetics (τ, μs). Data represents the mean ± SD (n = 3–4). Symbols (*) represent significant differences from wild type within light acclimated states based on an un-paired t-test (p < 0.05).

We also observed changes in PSII chlorophyll *a* fluorescence parameters using a FIRe fluorometer in cultures grown in sinusoidal light conditions. These were determined *ex situ* immediately after removal from their light environment. There was a significant reduction in the effective quantum yield of PSII in *npq4lhcsr1* compared to wild type after dawn (Fig 5A). Functional antenna size (sigma_PSII_, A^2^ quantum^-1^) changed very little across the light period, with *npq4lhcsr1* having a significantly reduced functional antenna size at the beginning of the light period only (Fig 5B). The rate of Q_B_ re-oxidation were also determined from the kinetics of fluorescence relaxation following a multiple turnover flash (MTF). Wild type and *npq4lhcsr1* cultures showed identical rates of Q_B_ re-oxidation throughout the day but oxidation times were longer at dawn and dusk than throughout the rest of the illumination period (Fig 5C). In contrast with sinusoidal light conditions, cultures grown in 12 hours 2134 μmol photons m^-2^ s^-1^ constant light, 12 hours dark in a ePBR displayed no differences in the effective quantum yield and functional antenna size of PSII, and a significant increase in Q_B_ re-oxidation 3 hours after dawn (S5 Fig).

### Quantification of lipid peroxidation of wild type and *npq4lhcsr1* under continuous and sinusoidal light regimes

**Fig 6:**
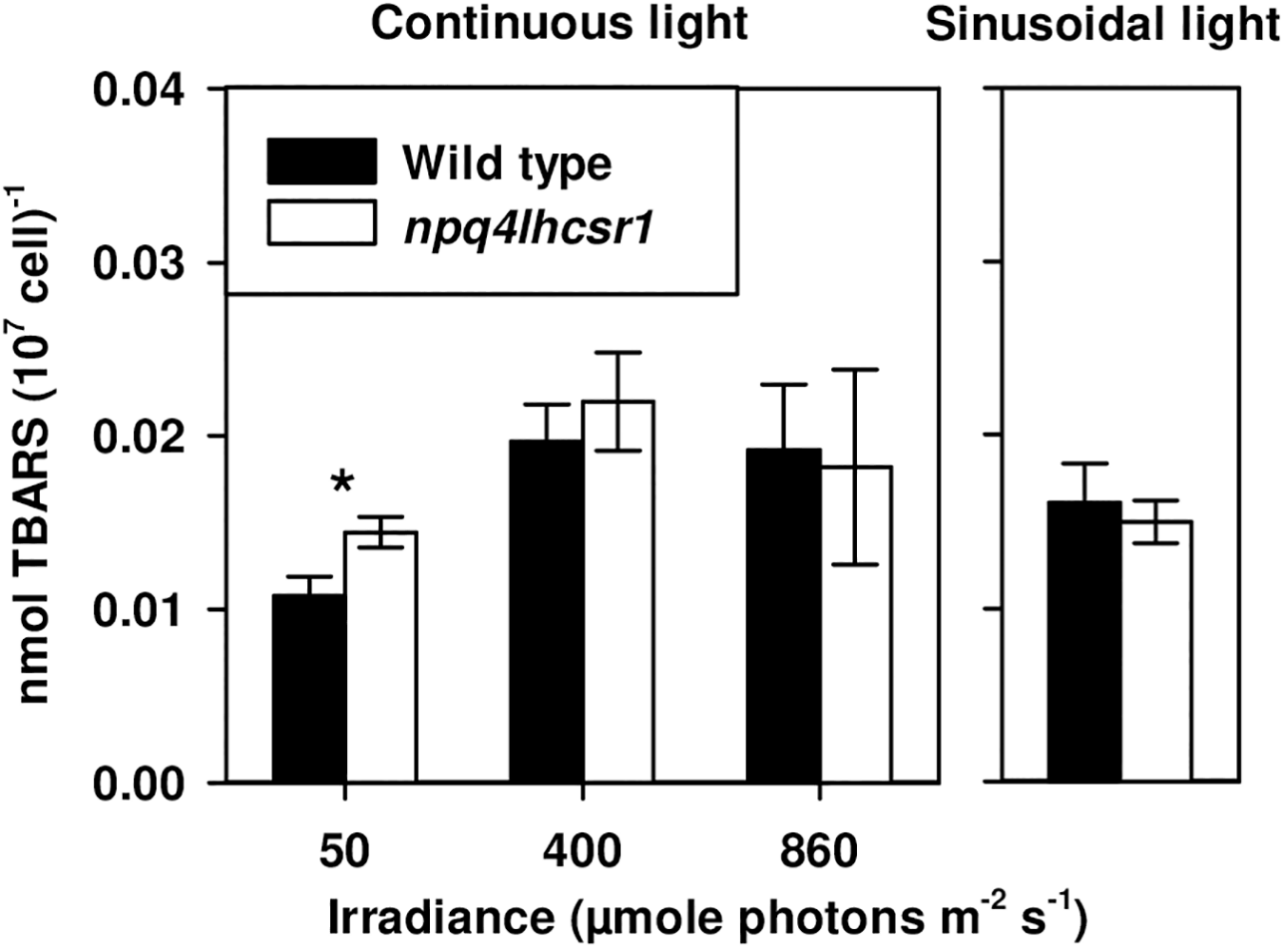
Thiobarbituric acid reactive substance (TBARS) concentrations per 10^7^ cell for wild type and *npq4lhcsr1* acclimated to different light regimes. Data represents the mean ± SD (n = 3–6). Symbols (*) represent significant differences from wild type within light acclimated states based on an un-paired t-test (p < 0.05).

Thiobarbituric reactive substances (TBARS) were used to quantify the extent of lipid peroxidation in cultures acclimated to continuous and sinusoidal light regimes. Lipid peroxidation occurs predominantly through a reaction between ^1^O_2_ and membrane lipids and has been used previously as a marker for reactive oxygen species (ROS) mediated stress in *Chlamydomonas* [45]. TBARS normalized per cell increased from 50 μmol photons m^-2^ s^-1^ to the higher light levels, including sinusoidal light (Fig 6). *npq4lhcsr1* had significantly higher TBARS than wild type only when grown at 50 μmol photons m^-2^ s^-1^ (Fig 6).

### Total organic carbon accumulation of wild type and *npq4lhcsr1* under a sinusoidal light regime

**Fig 7:**
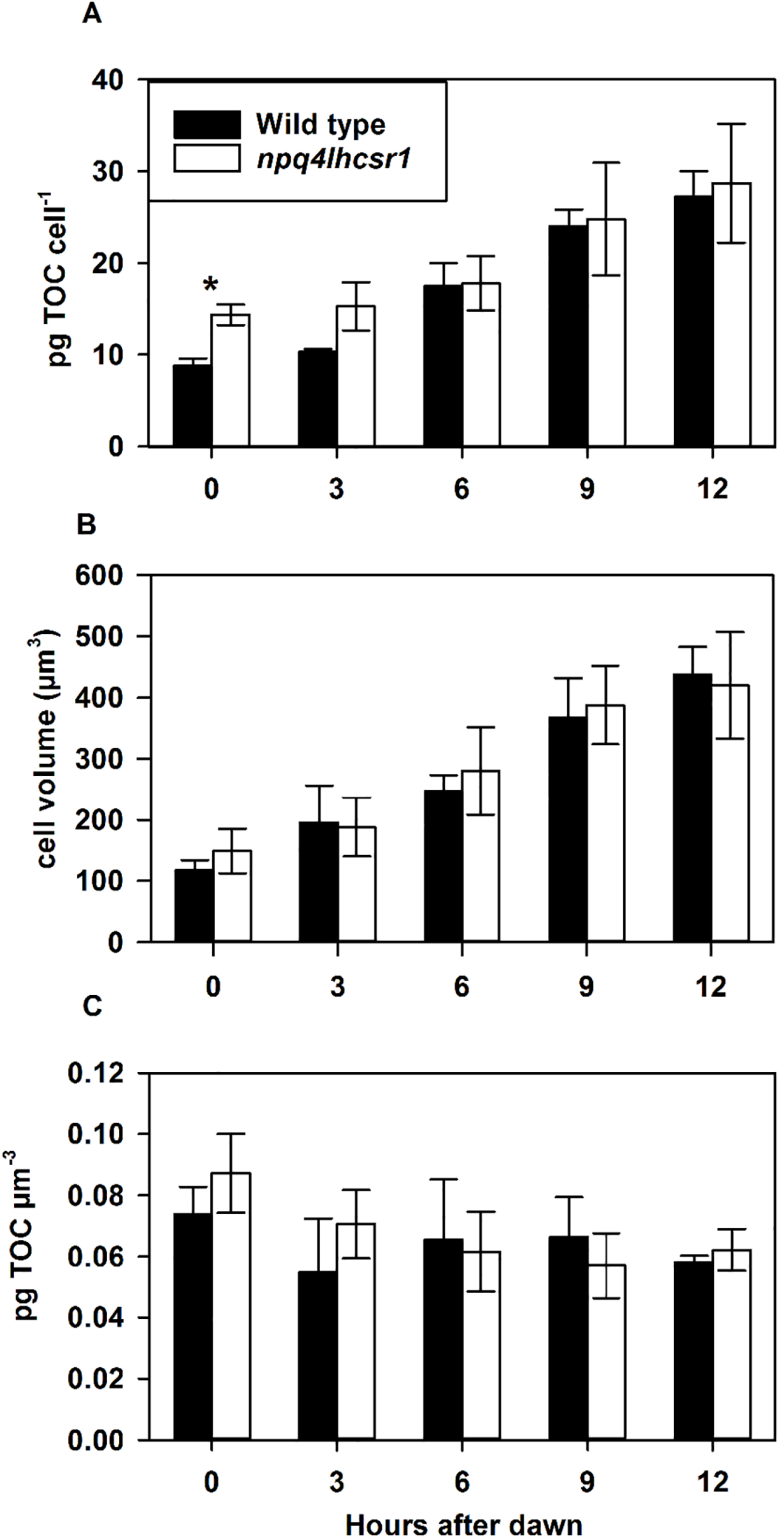
Total organic carbon (TOC) accumulation across a 12 hour sinusoidal light regime. (A) shows pg TOC cell^-1^, (B) shows Cell volume (μm^3^) of cells measured and (C) shows pg TOC normalized to cell volume. Data represents the mean ± SD (n = 5). Symbols (*) represent significant differences between wild type and *npq4lhcsr1* for each time point based on an un-paired t-test (p < 0.05).

We measured total organic carbon (TOC) content and cell size during the light period to determine if the loss of qE impacted carbon accumulation. Wild type cultures had 8.6 ± 0.6 pg TOC cell^-1^ and an average cell volume of 118 ± 16 μm^3^ at dawn and ended the light period at 25.5 ± 2.8 pg TOC cell^-1^ and an average cell volume of 438 ± 45 μm^3^ (Fig 7A and 7B). *npq4lhcsr1* cultures had significantly more TOC (12.8 ± 2.5 pg TOC cell^-1^) and a similar cell volume to wild type cultures (149 ± 37 μm^3^) at dawn, but ended the light period at approximately the same pg TOC cell^-1^ (26.1 ± 5.9 pg TOC cell^-1^) and cell volume (420 ± 87 μm^3^) as wild type (Fig 7A and 7B). However, there was no statistical difference in total carbon accumulated over the course of the day between the two strains (16.8 ± 2.3, and 13.3 ± 4.6 pg TOC cell^-1^, for wild type and *npqlhcsr1*, respectively; p > 0.05).

### Patterns of cell division

**Fig 8:**
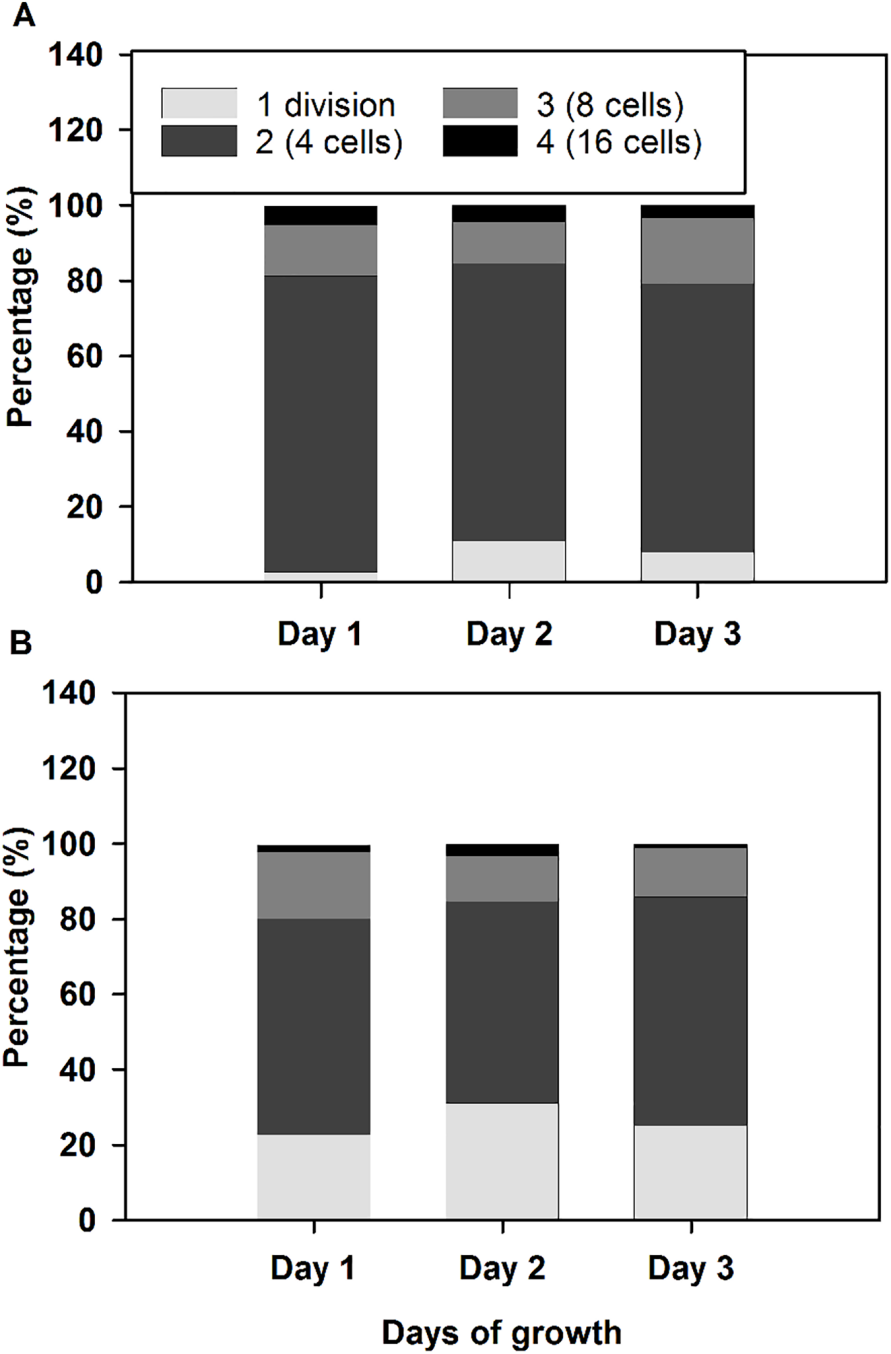
Distribution of cell divisions per progenitor cell during growth in sinusoidal light conditions. Cell division number was quantified for 3 consecutive days in single replicate wild type (A) and *npq4lhcr1* (B) cultures. Wild type cells averaged 2.2 divisions per night and *npq4lhcsr1* averaged 1.9 divisions per night across the three nights measured.

We did not observe any significant cell divisions for either strain during the light period in our sinusoidal cultures (S2 Fig). We assayed the number of cell fission events during three consecutive nights in wild type and *npq4lhcsr1* and observed that wild type underwent a greater number of multiple fission events per cell than *npq4lhcsr1* (Fig 8). This indicates that the reduced growth phenotype observed is due to a reduction in division number per cell at night.

## Discussion

LHCSR is required for qE in green algae, diatoms and presumably all algae with an LHC-based light harvesting antenna [21, 53, 54]. Previous work on the qE deficient mutant, *npq4*, showed that absence of LHCSR3 leads to a 50% reduction in NPQ capacity and this capacity could be recovered by complementation [21]. This lead to the hypothesis that LHCSR3 and LHCSR1 both could mediate qE in *Chlamydomonas*. Characterization of the *npq4lhcsr1* mutant indicates that complete loss of all of the LHCSR proteins (Fig 1A) correlated with a lack of rapidly inducible and reversible NPQ (Fig 1B). This indicates that LHCSR3 and LHCSR1 proteins both contribute to qE and this result is corroborated in other recently published studies [22, 55, 56].

Previous studies in *Chlamydomonas* have dissected the sensitivity of NPQ deficient mutants to light stress [21–23, 25, 45, 55]. Observations of *npq4* have shown that qE is important for maintaining fitness during a single step increase in light intensity [21, 23]. However, *npq4* displayed no significant reduction in exponential growth under continuous irradiances of 325 μmol photons m^-2^ s^-1^ [21]. We found that an absence of qE was associated with a small, but significant reduction (11%) in exponential growth rates only when acclimated to a continuous irradiance of 860 μmol photons m^-2^ s^-1^ (Fig 2A). Analogous experiments on a qE-deficient, PsbS mutant of *Arabidopsis thaliana*, *npq4*, observed similarly weak phenotypes under high light acclimated laboratory conditions [57]. Interestingly, photoprotective pigment mutants of *Chlamydomonas* lacking lutein or zeaxanthin and lutein show reduced growth rates compared to wild type cells at moderate light intensities of 350 μmol photons m^-2^ s^-1^ [25]. This may be due to their dual role in directly quenching excitation energy [27] and also their ability to quench ^1^O_2_ in the thylakoid membrane and reduce oxidative stress [3].

### Photoacclimation to steady-state excess light with and without qE

Our wild type light acclimation phenotype mirrored previous observations in *Chlamydomonas* whereby total chlorophyll content per cell decreases with increasing irradiance and maximal capacity for photosynthesis increases as do growth rates (Tables 1 & 2) [21, 58]. Previous studies performed at 400 μmol photons m^-2^ s^-1^ constant light revealed a decrease in the PSI:PSII ratio and a change in the ratio of LHCs relative to low light [58]. We also acclimated our cultures to continuous light at 860 μmol photons m^-2^ s^-1^, which is in excess of the two aforementioned studies. In this condition, the chlorophyll *a*:*b* ratio of wild type cells was significantly increased relative to low light acclimated cultures. Chlorophyll *a* is only present in reaction centers and LHC proteins contain both chlorophylls *a* and *b* [1]. These results contribute to the suggestion that *Chlamydomonas* acclimates to excess light conditions by reducing the number of LHC proteins associated with each reaction center at very high light intensities. We did not directly investigate changes in protein composition during steady state growth.

The *npq4lhcsr1* produces more singlet oxygen compared to wild type cells, following a shift from 400 to 1600 μmol photons m^-2^ s^-1^ [22]. This increase in reactive oxygen species generation explains the observed decrease in *F*_*v*_*/F*_*m*_ over several days growth in excess light reported in the same study. We found that *F*_*v*_*/F*_*m*_ was also lower in *npq4lhcsr1* compared to the wild type in cells grown in 400 and 860 μmol photons m^-2^ s^-1^, suggesting that even fully acclimated cells experience photodamage due to a lack of qE. Despite this clear inhibition of PSII, we did not observe major changes in the photosynthetic capacity at low light (α) and irradiance at photosynthetic saturation (I_k_), regardless of growth irradiance. We did observe a slight, but statistically significant reduction in P_max_ at 860 μmol photons m^-2^ s^-1^ during short term assays (Fig 3 and Table 2). Additionally, we found no difference in the ability of cells to maintain open reaction centers during the *ex situ* assay of photosynthetic capability (1-qL, Fig 3). The sum of these results suggest that, while a lack of qE certainly affects light harvesting at the level of PSII, there is minimal detriment to the overall process of photosynthesis during growth in constant light.

The rest of our discussion relates to our novel observations of photophysiology in a changing light environment.

### Lack of qE results in distinct responses to sinusoidal light

The absence of LHCSR appears to alter the photosynthetic apparatus when cells are grown in a changing, sinusoidal light regime. The following observations were all made 6 hours into the light period, corresponding to the highest light intensity of the day. *npq4lhcsr1* displayed a large increase in P_max_ and α was 71% relative to wild type when normalized by cell. The mutant appeared to have an increase in the PSII:PSI ratio relative to wild type, as estimated by changes in the relative abundance of the D2 and PsaC proteins, respectively.

Decreases in the chlorophyll *a*:*b* ratio suggested an increase in the relative content of antenna relative to the photosystems if LHCSR was absent. However, this is a bulk measurement that could be associated with either photosystem. We also observed an increase in the relative abundance of D2 in *npq4lhcsr1*. This, in conjunction with no differences observed in PSII antenna protein Lhcb2, suggests a decrease in the antenna size of PSII in the mutant. But we note the Lhcb2 antibody was designed for use in *Arabidopsis* and while it recognizes LHCs in *Chlamydomonas*, it may recognize LHCs other than those associated with PSII. Estimations of the functional antenna size measurements using FIRe fluorescence techniques found no significant difference between wild type and *npq4lhcsr1* at midday (Fig 5B). Cumulatively, these observations indicate that absence of LHCSR impacts photoacclimation and likely alters the architecture of the light harvesting apparatus.

### Alternate energy dissipation strategies may compensate for the loss of qE in sinusoidal light

If excess light energy is unable to be dissipated as heat when light fluxes increase, then the plastoquinone pool may become more reduced at lower light fluxes in *npq4lhcsr1* compared to the wild type strain. To estimate the redox state of the PQ pool, we looked at the proportion of closed PSII reaction centers during rapid light curves (1-qL, [40]). We found that *npq4lhcsr1* cells maintained a PQ pool with a redox state comparable to wild type cells during a rapid light curve protocol. This could be due to an increased flux of electrons through alternate electron transport pathways mediated by the plastid terminal oxidase (PTOX, [59]), the mitochondrial Alternative Oxidase (AOX, [60]), or the Mehler reaction [61]. These mechanisms are known to be important for reducing the occurrence of oxidative damage during stress.

Alternately, *npq4lhcsr1* may maintain an equivalent PQ redox state to wild type by altering its LHCSR independent NPQ. qT capacity, which can quench excess energy by facilitating LHCII aggregation [17, 18], may be increased in *npq4lhcsr1*, but the lack of NPQ relaxation observed on filtered samples exposed to far red light does not support this hypothesis (S1 Fig). qZ or qI may also compensate for loss of qE in *npq4lhcsr1*. qZ takes tens of minutes to relax and occurs through the pH dependent accumulation of zeaxanthin in the thylakoid membranes antenna [14]. qI reflects quenching due to photodamage to the PSII D1 protein and can take greater than 30 minutes for full recovery [11]. In our sinusoidal light experiment, we observed minor reductions in *F*_*v*_*/F*_*m*_ (~0.6 after 6 hours in the light, vs. ~0.7 at dawn), so we cannot rule out that qZ or qI plays a role in quenching excess light energy in the mutant (Fig 5A). Neither zeaxanthin accumulation nor D1 damage and repair rates were measured in this study.

Photosynthetic cells can also utilize energy that is in excess of that used for growth by funneling reduced carbon to storage metabolites like starch or triacylglycerol [62, 63]. Reducing the capacity to make or utilize these energy storage compounds can result in increased photoinhibition in plants, suggesting a strong interaction between light harvesting and central metabolism [64]. We observed no change in the accumulation of organic carbon over the course of the day (16.8 ± 2.3, and 13.3 ± 4.6 pg TOC cell^-1^, for wild type and *npqlhcsr1*, respectively; p > 0.05). This shows that the relatively high P_max_ observed in the *npq4lhcsr1* mutant in the short term rapid light curve assay does not translate to more biomass. However, we only tested photosynthetic capacity at solar noon, so there may be other changes in photosynthetic efficiency that occur throughout the day that are not captured in our observations. It also suggests that relatively low levels of PSII photoinhibition observed by fluorescence techniques did not impact overall biomass accumulation during the day. This may not be surprising because the Rubisco activity of cells, and not light harvesting capacity, likely sets the maximal rates of carbon fixation during excess light [65].

### Potential costs associated with no qE

Based on the reduced *F*_*v*_*/F*_*m*_ observed in *npq4lhcsr1* relative to wild type, we conclude that damage to PSII reaction centers occurred (Table 2, Fig 5A). Degradation and re-synthesis of photo damaged D1 protein is an ATP dependent process [66–69]. Energetic costs associated with photoinhibition may be further increased due to the maintenance of a larger pool of amino acids and ribosomes for elongating the D1 polypeptides for insertion to PSII reaction centers [69]. To compensate for increased ATP demand associated with photoinhibition, *npq4lhcsr1* mutants may have higher relative rates of cyclic electron transport and mitochondrial respiration [63, 70, 71]. While cyclic electron transport was not measured in this study, dark respiration rates measured 6 hours after dawn were increased in the mutant compared to WT. This suggests that the absence of qE may increase the metabolic burden associated with repair to damaged cellular components.

We investigated if the reduction in the growth rates of *npq4lhscsr1* relative to wild type in sinusoidal conditions was due to changes in cell divisions at night. *Chlamydomonas* cells grown under day:night cycles display synchronous cell division, whereby organic carbon stores and cell size increases during the day (Fig 5) [72–74]. Cells then divide at night by multiple fission events (2^n^), where n is the number of cell divisions that occurs in each progenitor cell [73, 74]. We examined wild type and *npq4lhcsr1* cell division number across 3 days by assaying cell division number from an individual mother cell after dark incubation on plates [50]. Using this technique, we observed a higher proportion of *npq4lhcsr1* cells undergo single divisions relative to wild type (Fig 8). This observation, coupled with our biomass accumulation results, indicates that the reduced growth rate phenotype observed is not due to a reduced capacity to fix carbon, but instead because of a reduction in cell divisions at night. Additionally, we observed no changes in growth rate between the wild type and mutant when cells were grown at 2134 μmol photons m^-2^ s^-1^ on a 12 hour day:night cycle (S4 Fig). This suggests that there is a metabolic penalty associated with an inability to perform qE under sinusoidal light conditions. Our measurements of respiration at noon suggest that there is a higher metabolic rate per cell in the mutant compared to the wild type (Table 2), but this increase in respiration was not consistently observed at night (S3 Fig). More energy could be required to repair macromolecules damaged by oxidative stress, but our measurements of TBARS suggest that there is no increase in peroxidated macromolecules at noon (Fig 6). However, this measurement of steady state damage products does not take into account active repair processes or the production of additional defenses against oxidative stress such as antioxidant enzymes and small molecules [75]. Maintaining these enzymes, antioxidants and the affiliated machinery to produce them (as discussed above for D1 replacement) may also reduce the amount of carbon and energy available for cell divisions at night.

In summary, the results from our sinusoidal light experiments show that a lack of qE capacity in *Chlamydomonas* leads to a reduction in daily growth rates, but this is not due to a decrease in carbon accumulation during the day. However, there is some metabolic penalty associated with no qE which leads to reduced cell division rates at night. The *Arabidopsis thaliana* qE-less *npq4* mutant grown in the field had a lower seed production compared to wild type, but similar vegetative biomass production [76]. These results highlight the importance for assaying the physiology of photosynthetic mutants in more realistic environments to determine the true impact of a given process on growth and survival.

### Engineering NPQ to increase photosynthetic productivites in mass culture

Algae growing in dense culture experience rapid changes in the light environment that are superimposed on the daily sinusoidal light cycle [77]. The amount of time spent in the dark or sub-saturating light levels relative to the amount of time spent in excess light influences the photoacclimation process and NPQ induction. Improving photosynthetic efficiency in response to their growth conditions has been a major goal of algal bioengineering [78, 79]. *Chlamydomonas* cells spending more time in the dark show signs of low light photoacclimation and accumulate less LHCSR3 relative to those grown in scenarios with longer periods of excess light or in constant light [80]. Cells acclimated to high density and fluctuating light conditions also exhibited lower biomass productivities, which was presumed to be due to the increase in energy required to repair photodamage [80]. These results suggested that high light photo-acclimation responses such as the induction of qE capacity are required to maintain high productivities in industrial situations.

However, the down regulation or absence of light energy dissipation pathways in algae and cyanobacteria may result in higher biomass productivities overall. It has been hypothesized that the slow reversal of NPQ during the transition to low light conditions may reduce the conversion efficiency of photons to biomass. So, it may be that reducing NPQ capacity may result in more biomass accumulation [77]. Indeed, Peers (2015) found that the deletion of the OCP protein, responsible for NPQ in the phycobillisome of many cyanobacteria, resulted in a 30% increase of biomass accumulation in dense cultures of *Synechocystis* vs. wild type cells in conditions that mimicked outdoor light environments [81]. Furthermore, it appeared that the *npq4* strain of *Chlamydomonas* also accumulated more biomass not only in constant moderate light (200–800 μmol photons m^-2^ s^-1^) but also in rapidly oscillating light relative to wild type cells [22]. This difference was not observed in the mutant *npq4lhcsr1* strain grown at 200–400 μmol photons m^-2^ s^-1^ constant light [22]. We highlight that these experiments on *Chlamydomonas* were carried out without a day:night cycle and therefore reduction in growth associated with nighttime divisions would not have been observed.

We observed no significant differences in biomass accumulation during the day between wild type and *npq4lhcsr1* cells in our sinusoidal light conditions (Fig 7A), but an overall reduced growth rate across days due to fewer nightly cell divisions (Figs 2A and 8). We note that our conditions resemble a low biomass photobioreactor as significant amounts of light still penetrated the entire culture (Fig 2B, [38, 39]). The relative productivities could change with increasing cell density, mixing rates or nutrient status, which can affect cell physiology or light distribution. Our observations suggest that manipulating the kinetics or capacity of NPQ may be a promising strategy for improving photosynthetic efficiency in algae and plants [78, 82, 83], but the complex environment associated with production conditions may negate promising predictions based off experiments done in simple lab environments [77].

## Supporting information

S1 Fig NPQ of chlorophyll fluorescence and 1-qL for wild type and *npq4lhcsr1* acclimated to different light levels. Wild type (closed circles) and the *npq4lhcsr1* mutant (open squares) acclimated to either 50 (A-D), 400 (E-H) and 860 μmol photons m^-2-^ s^-1^ (I-L) were exposed to an actinic light level of 600 (A, E, I, B, F, J) or 2005 (C, G, K, D, H, L) μmol photons m^-2-^ s^-1^ (white bars) followed by 10 minutes darkness (black bar) and far red light illumination to re-associate LHCII with PSII (state 1 transition) by preferentially driving PSI charge separation. Data represent means ± s.d. (n = 3).
(TIF)

S2 Fig Cell densities used for total organic carbon (TOC) measurements. (A) Average cell densities for wild type and *npq4lhcsr1* across a single day (n = 5). (B) Individual measurements for wild type and *npq4lhcsr1*.
(TIF)

S3 Fig Respiration rates at night in a sinusoidal light regime. Respiration rates in wild type (black circles) and *npq4lhcsr1* (open squares) were measured immediately after sampling at 20 minutes before and after the night period and 0.5, 1.5, 3, 6, 9, 10.5 and 11.5 hour after dusk. Data represents the mean ± SD (n = 3). Symbols (*) represent significant differences between wild type and *npq4lhcsr1* for each time point based on an un-paired t-test (p < 0.05).
(TIF)

S4 Fig Growth rates in ePBRs under a constant 2134 μmol photons m^-2-^ s^-1^ on a 12 hour day:Night light regime. (A) Natural log of cell densities across 2 days of growth for biological replicates (A-C) of wild type and *npq4lhcsr1*.(B) Growth rate per day. Data in B represents the mean ± SD (n = 3).
(TIF)

S5 Fig Photophysiology of photosystem II in ePPRs during growth in constant2134 μmol photons m^-2-^ s^-1^ on a 12 hour day:Night light regime. (A) Fv/Fm (B) Functional antenna size (sigma_PSII_, A^2^ quantum^-1^). Q_B_ re-oxidation kinetics (τ, μs). Data represents the mean ± SD (n = 3). Symbols (*) represent significant differences from wild type within each timepoint based on an un-paired t-test (p < 0.05).
(TIF)

S1 File Cantrell peers supporting. Excel file containing original data associated with this manuscript.
(XLSX)

## References

[1] Falkowski PG, Raven JA. Aquatic Photosynthesis. Princeton University Press 2013.

[2] Jallet D, Cantrell M, Peers G. Chloroplasts: Current research and future trends Kirchhoff H, editor: Caister Academic Press; 2016.

[3] Triantaphylides C, Havaux M. Singlet oxygen in plants: production, detoxification and signaling. Trends Plant Sci. 2009;14(4):219–28. doi: 10.1016/j.tplants.2009.01.008 . 19303348 10.1016/j.tplants.2009.01.008

[4] Krieger-Liszkay A, Fufezan C, Trebst A. Singlet oxygen production in photosystem II and related protection mechanism. Photosynth Res. 2008;98(1–3):551–64. doi: 10.1007/s11120-008-9349-3 . 18780159 10.1007/s11120-008-9349-3

[5] Derks A, Schaven K, Bruce D. Diverse mechanisms for photoprotection in photosynthesis. Dynamic regulation of photosystem II excitation in response to rapid environmental change. Biochim Biophys Acta. 2015;1847(4–5):468–85. doi: 10.1016/j.bbabio.2015.02.008 . 25687894 10.1016/j.bbabio.2015.02.008

[6] Erickson E, Wakao S, Niyogi KK. Light stress and photoprotection in Chlamydomonas reinhardtii. Plant J. 2015;82(3):449–65. doi: 10.1111/tpj.12825 . 25758978 10.1111/tpj.12825

[7] Ruban AV. Evolution under the sun: optimizing light harvesting in photosynthesis. J Exp Bot. 2015;66(1):7–23. doi: 10.1093/jxb/eru400 . 25336689 10.1093/jxb/eru400

[8] Wobbe L, Bassi R, Kruse O. Multi-Level Light Capture Control in Plants and Green Algae. Trends Plant Sci. 2016;21(1):55–68. doi: 10.1016/j.tplants.2015.10.004 . 26545578 10.1016/j.tplants.2015.10.004

[9] Tyystjarvi E, Aro EM. The rate constant of photoinhibition, measured in lincomycin-treated leaves, is directly proportional to light intensity. Proc Natl Acad Sci U S A. 1996;93(5):2213–8. doi: 10.1073/pnas.93.5.2213 11607639 10.1073/pnas.93.5.2213 PMC39937

[10] Aro EM, Virgin I, Andersson B. Photoinhibition of Photosystem II. Inactivation, protein damage and turnover. Biochim Biophys Acta. 1993;1143(2):113–34. doi: 10.1016/0005-2728(93)90134-2 . 8318516 10.1016/0005-2728(93)90134-2

[11] Takahashi S, Murata N. Interruption of the Calvin cycle inhibits the repair of Photosystem II from photodamage. Biochim Biophys Acta. 2005;1708(3):352–61. doi: 10.1016/j.bbabio.2005.04.003 . 15955527 10.1016/j.bbabio.2005.04.003

[12] Wehner A, Grasses T, Jahns P. De-epoxidation of violaxanthin in the minor antenna proteins of photosystem II, LHCB4, LHCB5, and LHCB6. J Biol Chem. 2006;281(31):21924–33. doi: 10.1074/jbc.M602915200 . 16754673 10.1074/jbc.M602915200

[13] Nilkens M, Kress E, Lambrev P, Miloslavina Y, Muller M, Holzwarth AR, et al Identification of a slowly inducible zeaxanthin-dependent component of non-photochemical quenching of chlorophyll fluorescence generated under steady-state conditions in Arabidopsis. Biochim Biophys Acta. 2010;1797(4):466–75. doi: 10.1016/j.bbabio.2010.01.001 . 20067757 10.1016/j.bbabio.2010.01.001

[14] Dall'Osto L, Caffarri S, Bassi R. A mechanism of nonphotochemical energy dissipation, independent from PsbS, revealed by a conformational change in the antenna protein CP26. Plant Cell. 2005;17(4):1217–32. doi: 10.1105/tpc.104.030601 . 15749754 10.1105/tpc.104.030601 PMC1087998

[15] Depege N, Bellafiore S, Rochaix JD. Role of chloroplast protein kinase Stt7 in LHCII phosphorylation and state transition in Chlamydomonas. Science. 2003;299(5612):1572–5. doi: 10.1126/science.1081397 . 12624266 10.1126/science.1081397

[16] Lemeille S, Willig A, Depege-Fargeix N, Delessert C, Bassi R, Rochaix JD. Analysis of the chloroplast protein kinase Stt7 during state transitions. PLoS Biol. 2009;7(3):e45 doi: 10.1371/journal.pbio.1000045 . 19260761 10.1371/journal.pbio.1000045 PMC2650728

[17] Nagy G, Unnep R, Zsiros O, Tokutsu R, Takizawa K, Porcar L, et al Chloroplast remodeling during state transitions in Chlamydomonas reinhardtii as revealed by noninvasive techniques in vivo. Proc Natl Acad Sci U S A. 2014;111(13):5042–7. doi: 10.1073/pnas.1322494111 . 24639515 10.1073/pnas.1322494111 PMC3977285

[18] Unlu C, Drop B, Croce R, van Amerongen H. State transitions in Chlamydomonas reinhardtii strongly modulate the functional size of photosystem II but not of photosystem I. Proc Natl Acad Sci U S A. 2014;111(9):3460–5. doi: 10.1073/pnas.1319164111 . 24550508 10.1073/pnas.1319164111 PMC3948275

[19] Niyogi KK, Bjorkman O, Grossman AR. Chlamydomonas Xanthophyll Cycle Mutants Identified by Video Imaging of Chlorophyll Fluorescence Quenching. Plant Cell. 1997;9(8):1369–80. doi: 10.1105/tpc.9.8.1369 . 12237386 10.1105/tpc.9.8.1369 PMC157004

[20] Horton P, Ruban AV, Walters RG. Regulation of Light Harvesting in Green Plants (Indication by Nonphotochemical Quenching of Chlorophyll Fluorescence). Plant Physiol. 1994;106(2):415–20. . 12232338 10.1104/pp.106.2.415 PMC159545

[21] Peers G, Truong TB, Ostendorf E, Busch A, Elrad D, Grossman AR, et al An ancient light-harvesting protein is critical for the regulation of algal photosynthesis. Nature. 2009;462(7272):518–21. doi: 10.1038/nature08587 . 19940928 10.1038/nature08587

[22] Berteotti S, Ballottari M, Bassi R. Increased biomass productivity in green algae by tuning non-photochemical quenching. Sci Rep. 2016;6:21339 doi: 10.1038/srep21339 . 26888481 10.1038/srep21339 PMC4758054

[23] Allorent G, Tokutsu R, Roach T, Peers G, Cardol P, Girard-Bascou J, et al A dual strategy to cope with high light in Chlamydomonas reinhardtii. Plant Cell. 2013;25(2):545–57. doi: 10.1105/tpc.112.108274 . 23424243 10.1105/tpc.112.108274 PMC3608777

[24] Tokutsu R, Minagawa J. Energy-dissipative supercomplex of photosystem II associated with LHCSR3 in Chlamydomonas reinhardtii. Proc Natl Acad Sci U S A. 2013;110(24):10016–21. doi: 10.1073/pnas.1222606110 . 23716695 10.1073/pnas.1222606110 PMC3683755

[25] Niyogi KK, Bjorkman O, Grossman AR. The roles of specific xanthophylls in photoprotection. Proc Natl Acad Sci U S A. 1997;94(25):14162–7. doi: 10.1073/pnas.94.25.14162 . 9391170 10.1073/pnas.94.25.14162 PMC28450

[26] Li Z, Peers G, Dent RM, Bai Y, Yang SY, Apel W, et al Evolution of an atypical de-epoxidase for photoprotection in the green lineage. Nat Plants. 2016;2:16140 doi: 10.1038/nplants.2016.140 . 27618685 10.1038/nplants.2016.140 PMC5021192

[27] Bonente G, Ballottari M, Truong TB, Morosinotto T, Ahn TK, Fleming GR, et al Analysis of LhcSR3, a protein essential for feedback de-excitation in the green alga Chlamydomonas reinhardtii. PLoS Biol. 2011;9(1):e1000577 doi: 10.1371/journal.pbio.1000577 . 21267060 10.1371/journal.pbio.1000577 PMC3022525

[28] Liguori N, Roy LM, Opacic M, Durand G, Croce R. Regulation of light harvesting in the green alga Chlamydomonas reinhardtii: the C-terminus of LHCSR is the knob of a dimmer switch. J Am Chem Soc. 2013;135(49):18339–42. doi: 10.1021/ja4107463 . 24261574 10.1021/ja4107463

[29] Ledford HK, Baroli I, Shin JW, Fischer BB, Eggen RI, Niyogi KK. Comparative profiling of lipid-soluble antioxidants and transcripts reveals two phases of photo-oxidative stress in a xanthophyll-deficient mutant of Chlamydomonas reinhardtii. Mol Genet Genomics. 2004;272(4):470–9. doi: 10.1007/s00438-004-1078-5 . 15517390 10.1007/s00438-004-1078-5

[30] Brueggeman AJ, Gangadharaiah DS, Cserhati MF, Casero D, Weeks DP, Ladunga I. Activation of the carbon concentrating mechanism by CO2 deprivation coincides with massive transcriptional restructuring in Chlamydomonas reinhardtii. Plant Cell. 2012;24(5):1860–75. doi: 10.1105/tpc.111.093435 . 22634764 10.1105/tpc.111.093435 PMC3442574

[31] Yamano T, Miura K, Fukuzawa H. Expression analysis of genes associated with the induction of the carbon-concentrating mechanism in Chlamydomonas reinhardtii. Plant Physiol. 2008;147(1):340–54. doi: 10.1104/pp.107.114652 . 18322145 10.1104/pp.107.114652 PMC2330288

[32] Zhang Z, Shrager J, Jain M, Chang CW, Vallon O, Grossman AR. Insights into the survival of Chlamydomonas reinhardtii during sulfur starvation based on microarray analysis of gene expression. Eukaryot Cell. 2004;3(5):1331–48. doi: 10.1128/EC.3.5.1331-1348.2004 . 15470261 10.1128/EC.3.5.1331-1348.2004 PMC522608

[33] Naumann B, Busch A, Allmer J, Ostendorf E, Zeller M, Kirchhoff H, et al Comparative quantitative proteomics to investigate the remodeling of bioenergetic pathways under iron deficiency in Chlamydomonas reinhardtii. Proteomics. 2007;7(21):3964–79. doi: 10.1002/pmic.200700407 . 17922516 10.1002/pmic.200700407

[34] Ballottari M, Truong TB, De Re E, Erickson E, Stella GR, Fleming GR, et al Identification of pH-sensing Sites in the Light Harvesting Complex Stress-related 3 Protein Essential for Triggering Non-photochemical Quenching in Chlamydomonas reinhardtii. J Biol Chem. 2016;291(14):7334–46. doi: 10.1074/jbc.M115.704601 . 26817847 10.1074/jbc.M115.704601 PMC4817166

[35] Truong TB. Investigating the Role(s) of LHCSRs in Chlamydomonas reinhardtii. eScholarship University of California: UC Berkeley; 2011.

[36] McDuff RE, Chisholm SW. The calculation of in situ growth rates of phytoplankton populations from fractions of cells undergoing mitosis: A clarification1. Limnology and Oceanography. 1982;27(4):783–8. doi: 10.4319/lo.1982.27.4.0783

[37] Kropat J, Hong-Hermesdorf A, Casero D, Ent P, Castruita M, Pellegrini M, et al A revised mineral nutrient supplement increases biomass and growth rate in Chlamydomonas reinhardtii. Plant J. 2011;66(5):770–80. doi: 10.1111/j.1365-313X.2011.04537.x . 21309872 10.1111/j.1365-313X.2011.04537.x PMC3101321

[38] Lucker BF, Hall CC, Zegarac R, Kramer DM. The environmental photobioreactor (ePBR): An algal culturing platform for simulating dynamic natural environments. Algal Res. 2014;6:242–9. doi: 10.1016/j.algal.2013.12.007

[39] Jallet D, Caballero MA, Gallina AA, Youngblood M, Peers G. Photosynthetic physiology and biomass partitioning in the model diatom Phaeodactylum tricornutum grown in a sinusoidal light regime. Algal Res. 2016;18:51–60. doi: 10.1016/j.algal.2016.05.014

[40] Kramer DM, Johnson G, Kiirats O, Edwards GE. New Fluorescence Parameters for the Determination of QA Redox State and Excitation Energy Fluxes. Photosynth Res. 2004;79(2):209 doi: 10.1023/B:PRES.0000015391.99477.0d . 16228395 10.1023/B:PRES.0000015391.99477.0d

[41] Oxborough K, Baker NR. Resolving chlorophyll a fluorescence images of photosynthetic efficiency into photochemical and non-photochemical components—calculation of qP and Fv '/Fm ' without measuring Fo '. Photosynthesis Research. 1997;54(2):135–42. doi: 10.1023/A:1005936823310

[42] Ritchie RJ. Fitting light saturation curves measured using modulated fluorometry. Photosynth Res. 2008;96(3):201–15. doi: 10.1007/s11120-008-9300-7 . 18415696 10.1007/s11120-008-9300-7

[43] Platt T, Gallegos CL, Harrison WG. Photoinhibition of Photosynthesis in Natural Assemblages of Marine-Phytoplankton. J Mar Res. 1980;38(4):687–701.

[44] Kolber ZS, Prasil O, Falkowski PG. Measurements of variable chlorophyll fluorescence using fast repetition rate techniques: defining methodology and experimental protocols. Bba-Bioenergetics. 1998;1367(1–3):88–106. doi: 10.1016/S0005-2728(98)00135-2 9784616 10.1016/s0005-2728(98)00135-2

[45] Baroli I, Gutman BL, Ledford HK, Shin JW, Chin BL, Havaux M, et al Photo-oxidative stress in a xanthophyll-deficient mutant of Chlamydomonas. J Biol Chem. 2004;279(8):6337–44. doi: 10.1074/jbc.M312919200 . 14665619 10.1074/jbc.M312919200

[46] Hodges DM, DeLong JM, Forney CF, Prange RK. Improving the thiobarbituric acid-reactive-substances assay for estimating lipid peroxidation in plant tissues containing anthocyanin and other interfering compounds. Planta. 1999;207(4):604–11. doi: 10.1007/s004250050524 10.1007/s00425-017-2699-3 28456836

[47] Porra RJ. The chequered history of the development and use of simultaneous equations for the accurate determination of chlorophylls a and b. Photosynth Res. 2002;73(1–3):149–56. doi: 10.1023/A:1020470224740 . 16245116 10.1023/A:1020470224740

[48] Sun J, Liu DY. Geometric models for calculating cell biovolume and surface area for phytoplankton. J. Plankton Res. 2003;25(11):1331–46. doi: 10.1093/plankt/fbg096

[49] Shriwastav A, Mohmed J, Bose P, Shekhar M. Deconvoluting algal and bacterial biomass concentrations in algal-bacterial suspensions. J. Appl Phycol. 2014;27(1):211–22. doi: 10.1007/s10811-014-0302-x

[50] Umen JG, Goodenough UW. Control of cell division by a retinoblastoma protein homolog in Chlamydomonas. Genes Dev. 2001;15(13):1652–61. doi: 10.1101/gad.892101 . 11445540 10.1101/gad.892101 PMC312728

[51] Cross FR, Umen JG. The Chlamydomonas cell cycle. Plant J. 2015;82(3):370–92. doi: 10.1111/tpj.12795 . 25690512 10.1111/tpj.12795 PMC4409525

[52] Richard C, Ouellet H, Guertin M. Characterization of the LI818 polypeptide from the green unicellular alga Chlamydomonas reinhardtii. Plant Mol Biol. 2000;42(2):303–16. doi: 10.1023/a:1006340308077 . 10794530 10.1023/a:1006340308077

[53] Bailleul B, Rogato A, de Martino A, Coesel S, Cardol P, Bowler C, et al An atypical member of the light-harvesting complex stress-related protein family modulates diatom responses to light. Proc Natl Acad Sci U S A. 2010;107(42):18214–9. doi: 10.1073/pnas.1007703107 . 20921421 10.1073/pnas.1007703107 PMC2964204

[54] Niyogi KK, Truong TB. Evolution of flexible non-photochemical quenching mechanisms that regulate light harvesting in oxygenic photosynthesis. Curr Opin Plant Biol. 2013;16(3):307–14. doi: 10.1016/j.pbi.2013.03.011 . 23583332 10.1016/j.pbi.2013.03.011

[55] Correa-Galvis V, Redekop P, Guan K, Griess A, Truong TB, Wakao S, et al Photosystem II Subunit PsbS Is Involved in the Induction of LHCSR Protein-dependent Energy Dissipation in Chlamydomonas reinhardtii. J Biol Chem. 2016;291(33):17478–87. doi: 10.1074/jbc.M116.737312 . 27358399 10.1074/jbc.M116.737312 PMC5016143

[56] Dinc E, Tian L, Roy LM, Roth R, Goodenough U, Croce R. LHCSR1 induces a fast and reversible pH-dependent fluorescence quenching in LHCII in Chlamydomonas reinhardtii cells. Proc Natl Acad Sci U S A. 2016;113(27):7673–8. doi: 10.1073/pnas.1605380113 . 27335457 10.1073/pnas.1605380113 PMC4941471

[57] Golan T, Muller-Moule P, Niyogi KK. Photoprotection mutants of Arabidopsis thaliana acclimate to high light by increasing photosynthesis and specific antioxidants. Plant Cell Environ. 2006;29(5):879–87. doi: 10.1111/j.1365-3040.2005.01467.x . 17087471 10.1111/j.1365-3040.2005.01467.x

[58] Bonente G, Pippa S, Castellano S, Bassi R, Ballottari M. Acclimation of Chlamydomonas reinhardtii to different growth irradiances. J Biol Chem. 2012;287(8):5833–47. doi: 10.1074/jbc.M111.304279 . 22205699 10.1074/jbc.M111.304279 PMC3285353

[59] Houille-Vernes L, Rappaport F, Wollman FA, Alric J, Johnson X. Plastid terminal oxidase 2 (PTOX2) is the major oxidase involved in chlororespiration in Chlamydomonas. Proc Natl Acad Sci U S A. 2011;108(51):20820–5. doi: 10.1073/pnas.1110518109 ; PubMed Central PMCID: PMCPMC3251066. 22143777 10.1073/pnas.1110518109 PMC3251066

[60] Mathy G, Cardol P, Dinant M, Blomme A, Gerin S, Cloes M, et al Proteomic and functional characterization of a Chlamydomonas reinhardtii mutant lacking the mitochondrial alternative oxidase 1. J Proteome Res. 2010;9(6):2825–38. doi: 10.1021/pr900866e . 20408572 10.1021/pr900866e

[61] Asada K. THE WATER-WATER CYCLE IN CHLOROPLASTS: Scavenging of Active Oxygens and Dissipation of Excess Photons. Annu Rev Plant Physiol Plant Mol Biol. 1999;50:601–39. doi: 10.1146/annurev.arplant.50.1.601 . 15012221 10.1146/annurev.arplant.50.1.601

[62] Juergens MT, Disbrow B, Shachar-Hill Y. The Relationship of Triacylglycerol and Starch Accumulation to Carbon and Energy Flows during Nutrient Deprivation in Chlamydomonas reinhardtii. Plant Physiol. 2016;171(4):2445–57. doi: 10.1104/pp.16.00761 . 27325664 10.1104/pp.16.00761 PMC4972295

[63] Lucker B, Kramer DM. Regulation of cyclic electron flow in Chlamydomonas reinhardtii under fluctuating carbon availability. Photosynth Res. 2013;117(1–3):449–59. doi: 10.1007/s11120-013-9932-0 . 24113925 10.1007/s11120-013-9932-0

[64] Adams WW 3rd, Muller O, Cohu CM, Demmig-Adams B. May photoinhibition be a consequence, rather than a cause, of limited plant productivity? Photosynth Res. 2013;117(1–3):31–44. doi: 10.1007/s11120-013-9849-7 . 23695654 10.1007/s11120-013-9849-7

[65] Vandenhecke JM, Bastedo J, Cockshutt AM, Campbell DA, Huot Y. Changes in the Rubisco to photosystem ratio dominates photoacclimation across phytoplankton taxa. Photosynth Res. 2015;124(3):275–91. doi: 10.1007/s11120-015-0137-6 . 25862645 10.1007/s11120-015-0137-6

[66] Malnoe A, Wang F, Girard-Bascou J, Wollman FA, de Vitry C. Thylakoid FtsH protease contributes to photosystem II and cytochrome b6f remodeling in Chlamydomonas reinhardtii under stress conditions. Plant Cell. 2014;26(1):373–90. doi: 10.1105/tpc.113.120113 . 24449688 10.1105/tpc.113.120113 PMC3963582

[67] Nixon PJ, Michoux F, Yu J, Boehm M, Komenda J. Recent advances in understanding the assembly and repair of photosystem II. Ann Bot. 2010;106(1):1–16. doi: 10.1093/aob/mcq059 . 20338950 10.1093/aob/mcq059 PMC2889791

[68] Raven JA. Flight or flight: the Economics of Repair and Avoidance of Photoinhibition of Photosynthesis. Funct Ecol. 1989;3(1):5 doi: 10.2307/2389670

[69] Raven JA. The cost of photoinhibition. Physiol Plant. 2011;142(1):87–104. doi: 10.1111/j.1399-3054.2011.01465.x . 21382037 10.1111/j.1399-3054.2011.01465.x

[70] Alric J, Lavergne J, Rappaport F. Redox and ATP control of photosynthetic cyclic electron flow in Chlamydomonas reinhardtii (I) aerobic conditions. Biochimica et biophysica acta. 2010;1797:44–51. doi: 10.1016/j.bbabio.2009.07.009 . 19651104 10.1016/j.bbabio.2009.07.009

[71] Takahashi H, Clowez S, Wollman FA, Vallon O, Rappaport F. Cyclic electron flow is redox-controlled but independent of state transition. Nat Commun. 2013;4:1954 doi: 10.1038/ncomms2954 . 23760547 10.1038/ncomms2954 PMC3709502

[72] Zones JM, Blaby IK, Merchant SS, Umen JG. High-Resolution Profiling of a Synchronized Diurnal Transcriptome from Chlamydomonas reinhardtii Reveals Continuous Cell and Metabolic Differentiation. Plant Cell. 2015;27(10):2743–69. doi: 10.1105/tpc.15.00498 . 26432862 10.1105/tpc.15.00498 PMC4682324

[73] Craigie RA, Cavaliersmith T. Cell-Volume and the Control of the Chlamydomonas Cell-Cycle. J Cell Sci. 1982;54(Apr):173–91.

[74] Bisova K, Zachleder V. Cell-cycle regulation in green algae dividing by multiple fission. J Exp Bot. 2014;65(10):2585–602. doi: 10.1093/jxb/ert466 . 24441762 10.1093/jxb/ert466

[75] Apel K, Hirt H. Reactive oxygen species: metabolism, oxidative stress, and signal transduction. Annu Rev Plant Biol. 2004;55:373–99. doi: 10.1146/annurev.arplant.55.031903.141701 . 15377225 10.1146/annurev.arplant.55.031903.141701

[76] Kulheim C., Agren J. & Jansson S. Rapid regulation of light harvesting and plant fitness in the field. Science. 2002;297, 91–93. doi: 10.1126/science.1072359 12098696 10.1126/science.1072359

[77] Peers G. Increasing algal photosynthetic productivity by integrating ecophysiology with systems biology. Trends Biotechnol. 2014;32(11):551–5. doi: 10.1016/j.tibtech.2014.09.007 . 25306192 10.1016/j.tibtech.2014.09.007

[78] Murchie EH, Niyogi KK. Manipulation of photoprotection to improve plant photosynthesis. Plant Physiol. 2011;155(1):86–92. doi: 10.1104/pp.110.168831 . 21084435 10.1104/pp.110.168831 PMC3075776

[79] Perrine Z, Negi S, Sayre RT. Optimization of photosynthetic light energy utilization by microalgae. Algal Res. 2012;1(2):134–42. doi: 10.1016/j.algal.2012.07.002

[80] Yarnold J, Ross IL, Hankamer B. Photoacclimation and productivity of Chlamydomonas reinhardtii grown in fluctuating light regimes which simulate outdoor algal culture conditions. Algal Res. 2016;13:182–94. doi: 10.1016/j.algal.2015.11.001

[81] Peers G, inventorEnhancement of biomass production by disruption of light energy dissipation pathways. United States of America patent 8,940,508. 2015.

[82] Wobbe L, Remacle C. Improving the sunlight-to-biomass conversion efficiency in microalgal biofactories. J Biotechnol. 2015;201:28–42. doi: 10.1016/j.jbiotec.2014.08.021 . 25160918 10.1016/j.jbiotec.2014.08.021

[83] Horton P. Optimization of light harvesting and photoprotection: molecular mechanisms and physiological consequences. Philos Trans R Soc Lond B Biol Sci. 2012;367(1608):3455–65. doi: 10.1098/rstb.2012.0069 . 23148272 10.1098/rstb.2012.0069 PMC3497074

