# Supplementary figures and images for: A mutant of Chlamydomonas without LHCSR maintains high rates of photosynthesis, but has reduced cell division rates in sinusoidal light conditions
Source: PLoS One. 2017 Jun 23;12(6):e0179395. doi: 10.1371/journal.pone.0179395 (PMC5482440; doi:10.1371/journal.pone.0179395)

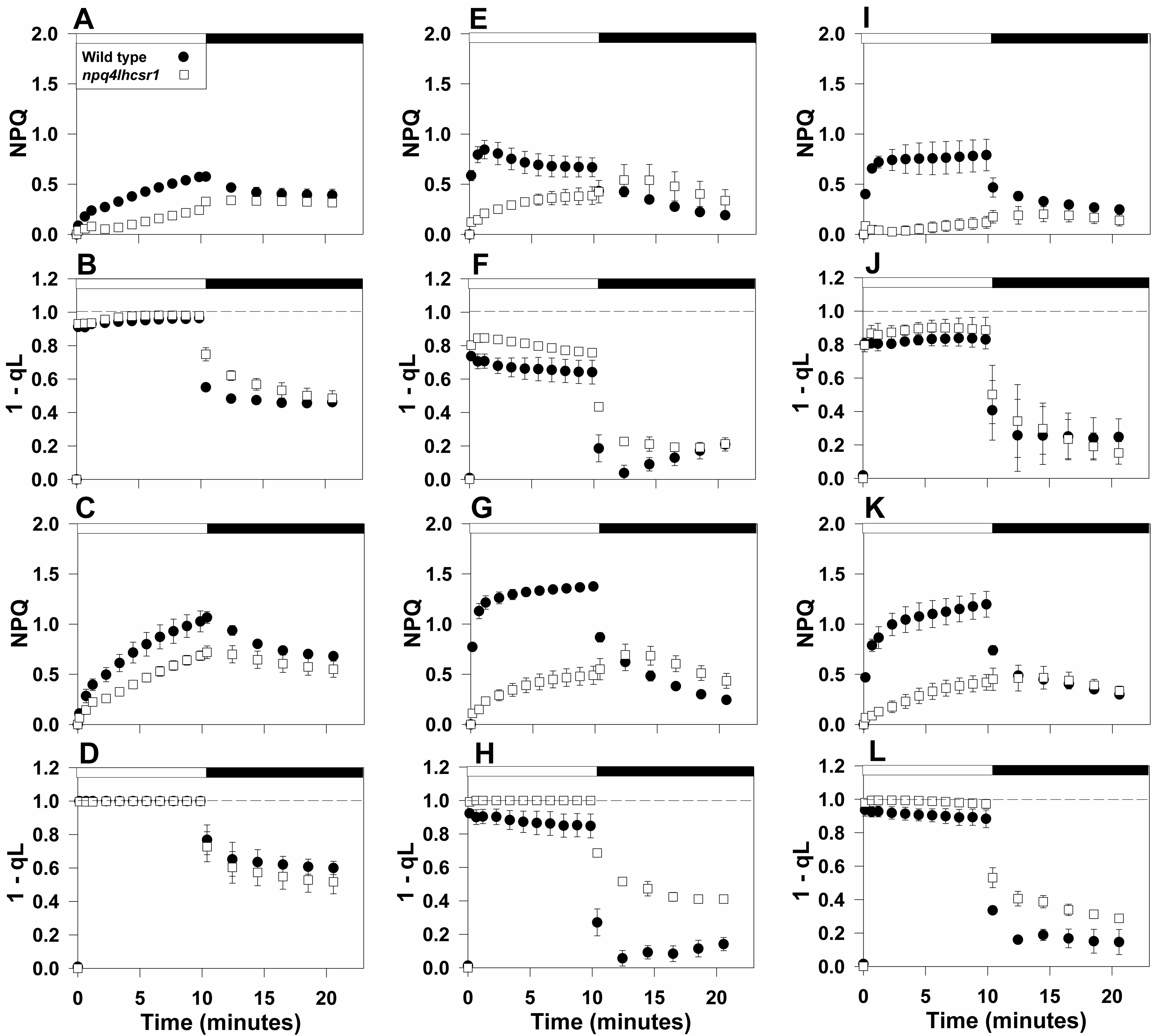

Supplement: S1 Fig — Wild type (closed circles) and the npq4lhcsr1 mutant (open squares) acclimated to either 50 (A-D), 400 (E-H) and 860 μmol photons m-2- s-1 (I-L) were exposed to an actinic light level of 600 (A, E, I, B, F, J) or 2005 (C, G, K, D, H, L) μmol photons m-2- s-1 (white bars) followed by 10 minutes darkness (black bar) and far red light illumination to re-associate LHCII with PSII (state 1 transition) by preferentially driving PSI charge separation. Data represent means ± s.d. (n = 3). (TIF) [file pone.0179395.s001.tif]

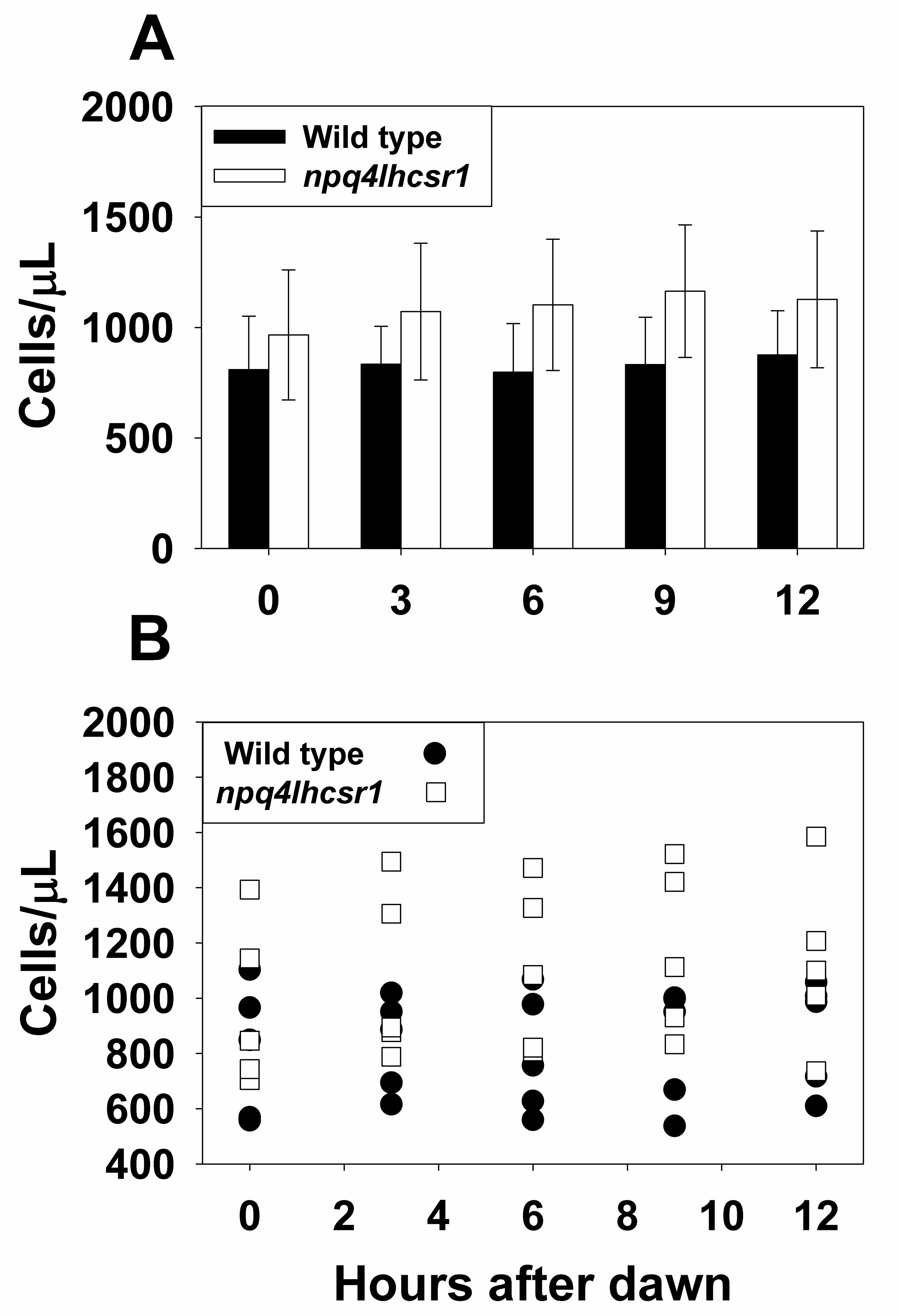

Supplement: S2 Fig — (A) Average cell densities for wild type and npq4lhcsr1 across a single day (n = 5). (B) Individual measurements for wild type and npq4lhcsr1. (TIF) [file pone.0179395.s002.tif]

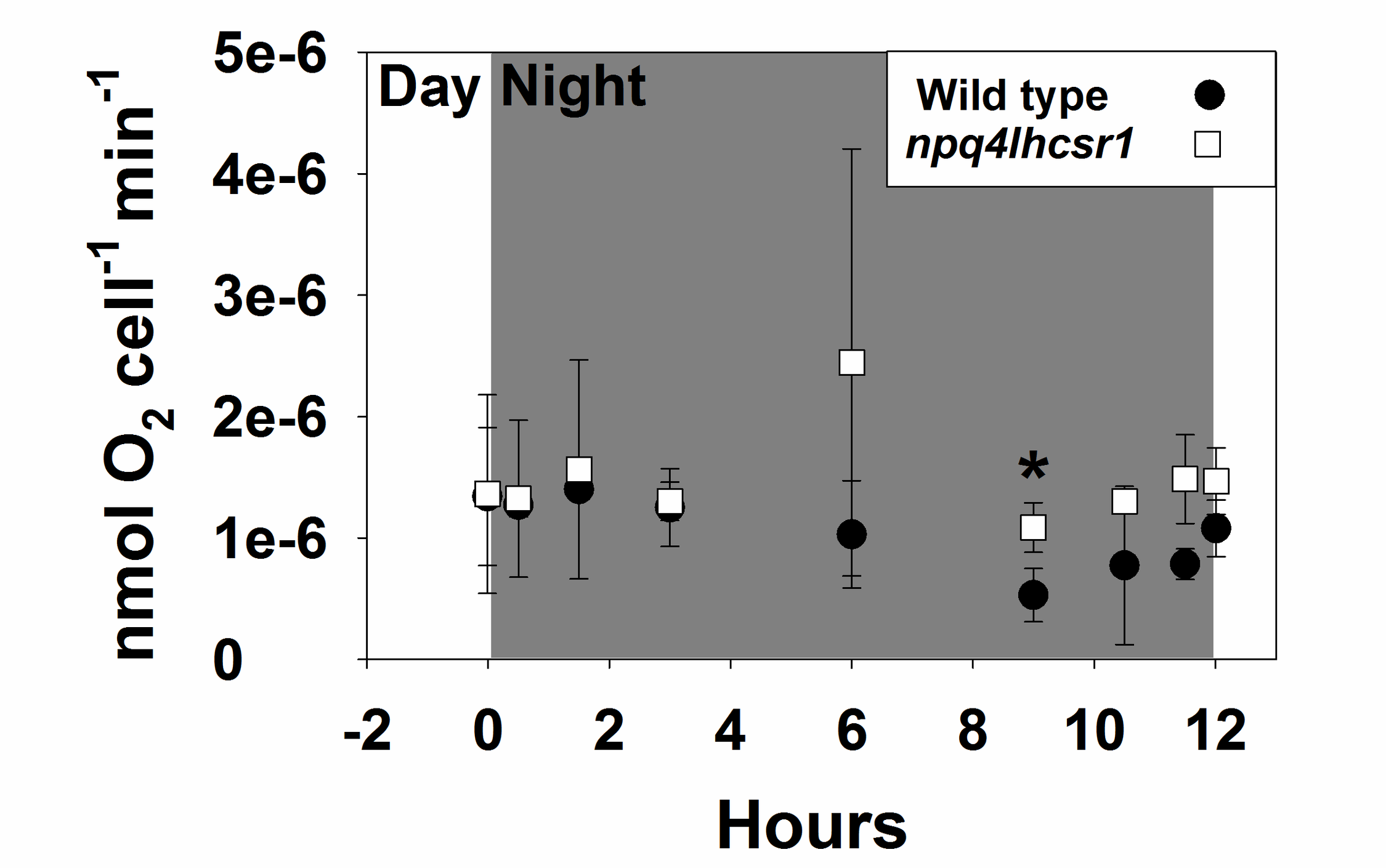

Supplement: S3 Fig — Respiration rates in wild type (black circles) and npq4lhcsr1 (open squares) were measured immediately after sampling at 20 minutes before and after the night period and 0.5, 1.5, 3, 6, 9, 10.5 and 11.5 hour after dusk. Data represents the mean ± SD (n = 3). Symbols (*) represent significant differences between wild type and npq4lhcsr1 for each time point based on an un-paired t-test (p < 0.05). (TIF) [file pone.0179395.s003.tif]

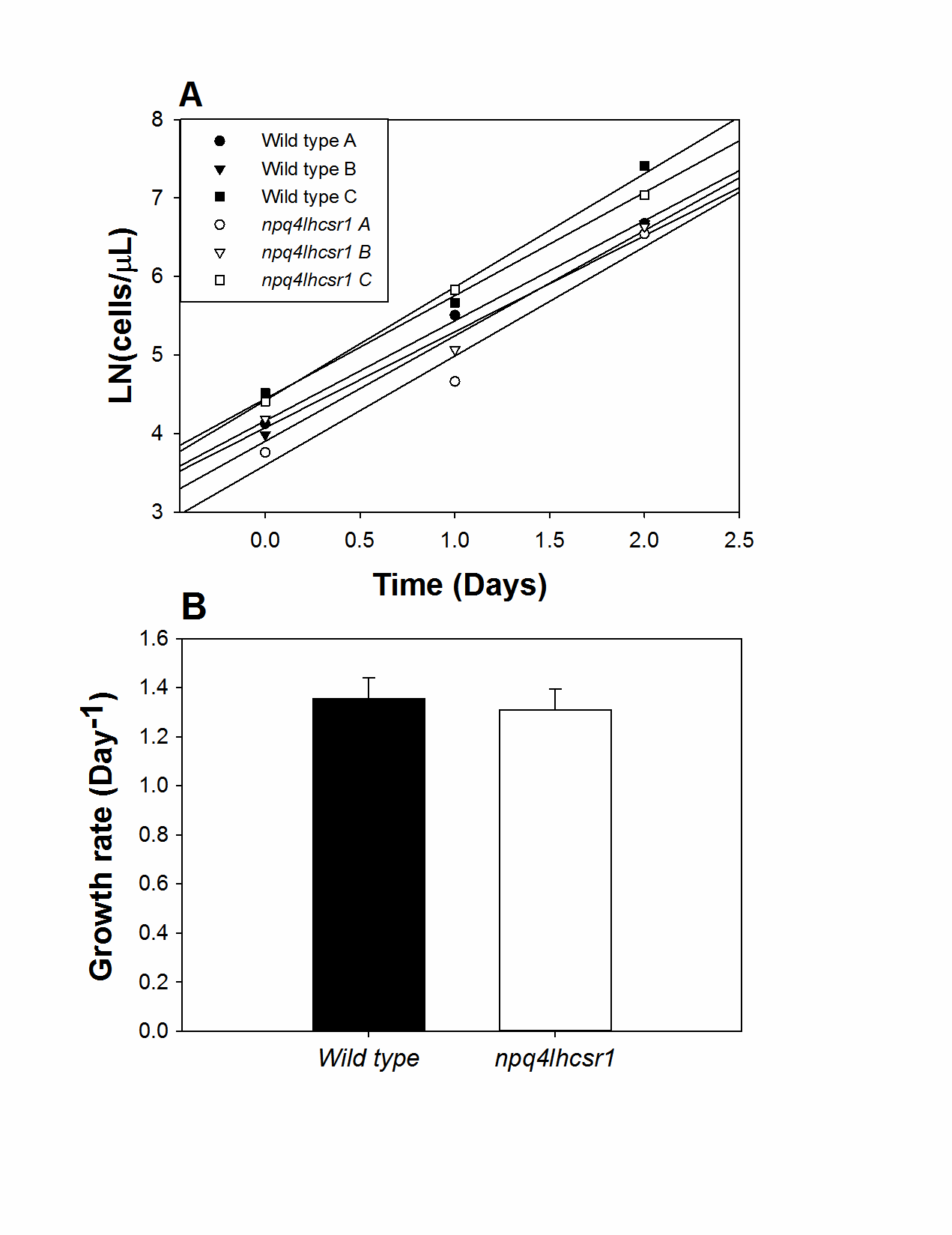

Supplement: S4 Fig — (A) Natural log of cell densities across 2 days of growth for biological replicates (A-C) of wild type and npq4lhcsr1.(B) Growth rate per day. Data in B represents the mean ± SD (n = 3). (TIF) [file pone.0179395.s004.tif]

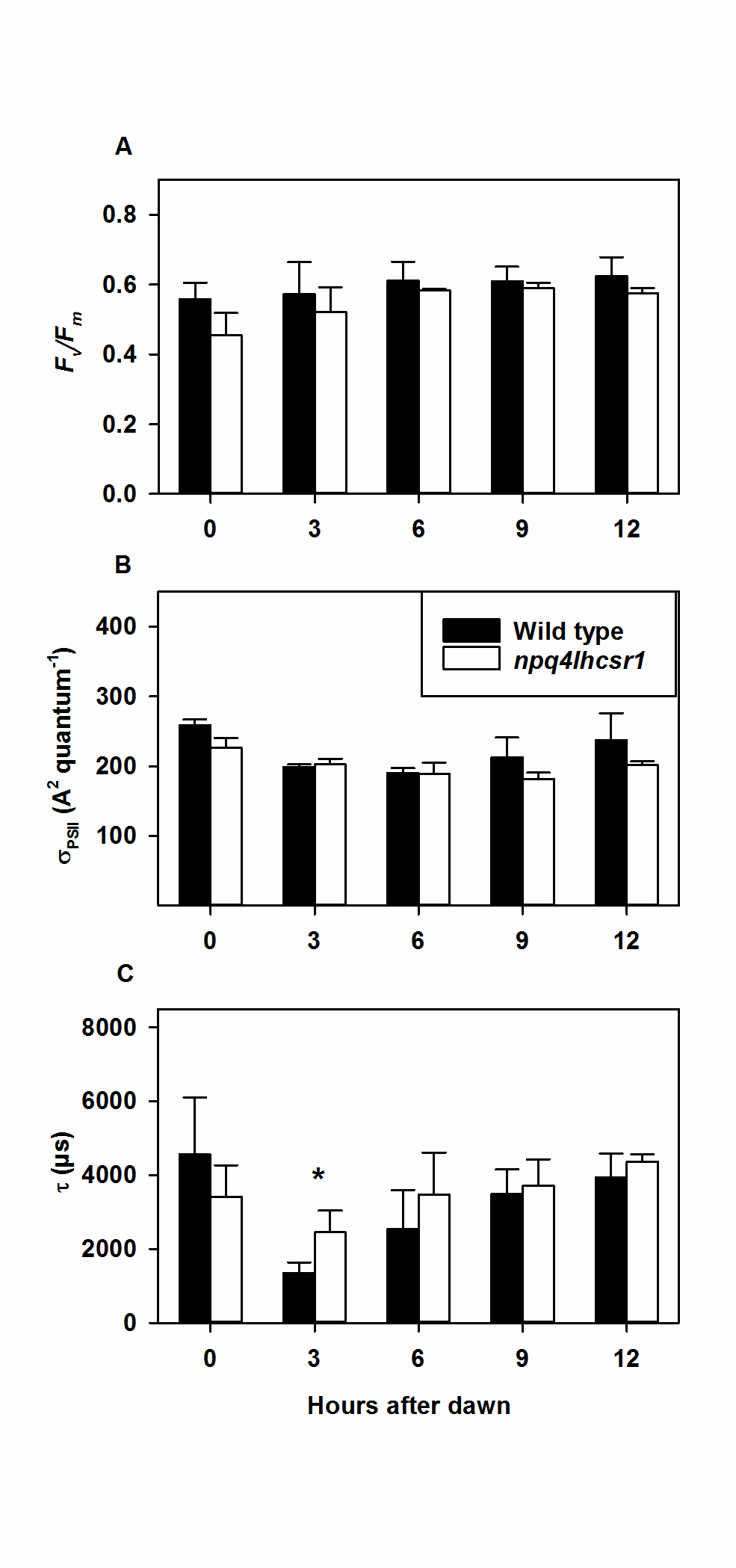

Supplement: S5 Fig — (A) Fv/Fm (B) Functional antenna size (sigmaPSII, A2 quantum-1). QB re-oxidation kinetics (τ, μs). Data represents the mean ± SD (n = 3). Symbols (*) represent significant differences from wild type within each timepoint based on an un-paired t-test (p < 0.05). (TIF) [file pone.0179395.s005.tif]
